# Synchronous Holocene thinning of Pine Island Glacier and its tributaries influenced by ice-shelf unpinning

**DOI:** 10.1038/s41467-026-76244-6

**Published:** 2026-08-11

**Authors:** Joanne S. Johnson, Sara C. Peters, Keir A. Nichols, Anna Ruth Halberstadt, Katie Brown, Dylan H. Rood, Klaus Wilcken, David Bett, Jonathan R. Adams, Greg Balco, John Woodward

**Affiliations:** 1 British Antarctic Survey, Cambridge, UK; 2 University of Texas at Austin, Austin, TX USA; 3 Imperial College London, London, UK; 4 Australian Nuclear Science and Technology Organisation (ANSTO), Lucas Heights, New South Wales, 2234 Australia; 5 Université de Lorraine, CNRS, CRPG, 54000, Nancy, France; 6 Lawrence Livermore National Laboratory, Livermore, CA 94551 USA; 7 Berkeley Geochronology Center, Berkeley, CA 94709 USA; 8 Northumbria University, Newcastle-upon-Tyne, UK; 9 Present Address: Scottish Universities Environmental Research Centre, University of Glasgow, Glasgow, UK; 10 Present Address: University College London, London, UK

**Keywords:** Climate sciences, Cryospheric science

## Abstract

Glaciers in Antarctica’s Amundsen Sea sector are retreating and thinning due to warm ocean inflow beneath ice shelves that buttress (restrain) glacier flow, but their future trajectory and implications for ice-mass loss remain uncertain. Here we present detailed cosmogenic-nuclide exposure-age data from the Hudson Mountains that document concerted thinning of Pine Island Glacier and its tributaries 8–5 ka. We contextualise this thinning using numerical ice-sheet modelling of the last deglaciation to link grounding-line dynamics and thinning characteristics. The model reconstructions attribute pauses in overall rapid thinning to ice-shelf pinning on bedrock highs, underscoring the importance of such features for ice-shelf stabilisation. Ice-flow paths from the sample sites all converge in the same grounding/calving-line region throughout the deglaciation, implying a common buttressing history and response to marine-grounding-line retreat that propagated upstream, controlling thinning. Our results support a key role for ice-shelf buttressing in influencing ice-sheet thinning in this region through the Holocene and into the future.

## Abstract (web summary)

Pine Island Glacier and its tributaries thinned synchronously 8000–5000 years ago, influenced by unpinning of its buttressing ice shelf from seabed highs. This finding suggests that continued ice-shelf weakening could trigger widespread inland ice loss.

## Introduction

**Fig. 1:**
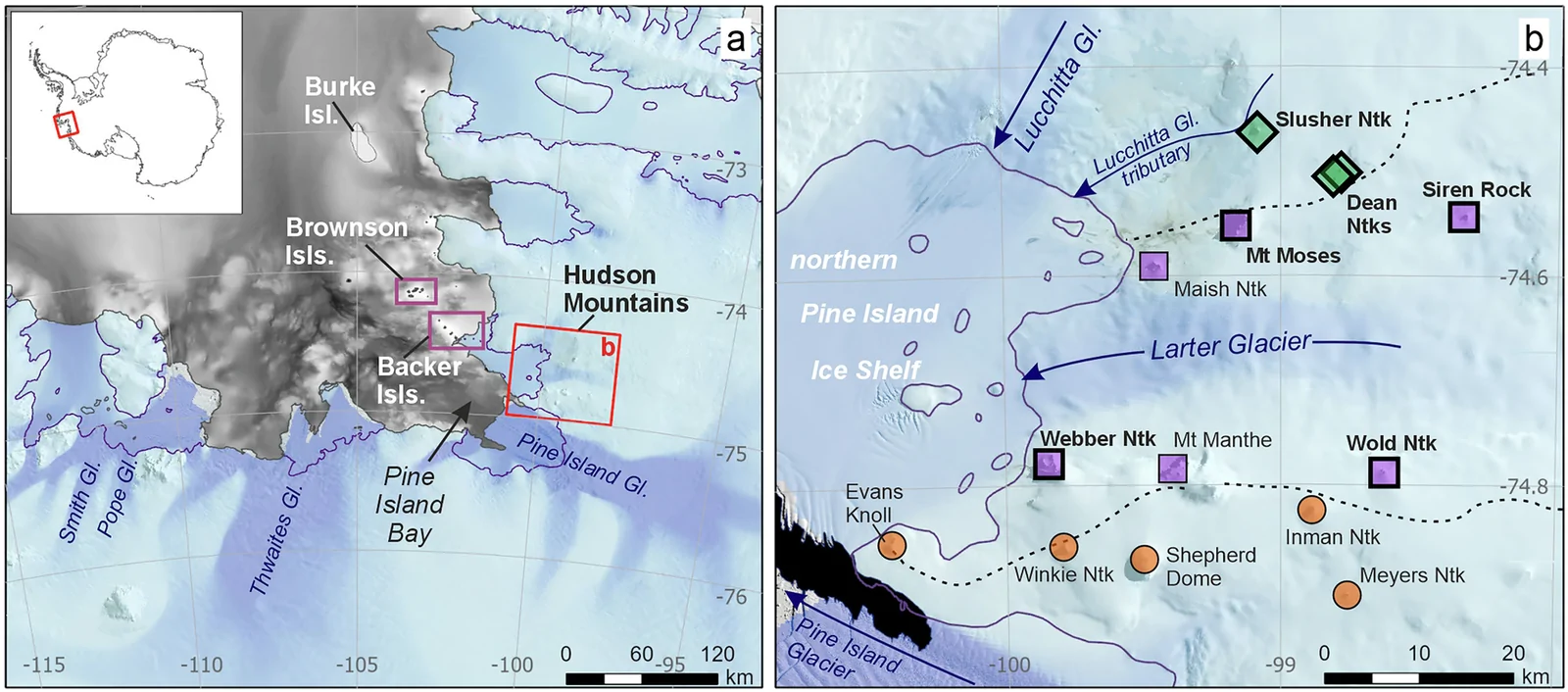
Location of study area in the Amundsen Sea sector of Antarctica. **a** Map of ice velocities and bathymetry^78^, with location of Hudson Mountains and Pine Island Bay shown alongside other places mentioned in the text. **b** Map of Hudson Mountains, showing nunatak sites sampled for exposure dating and location of Pine Island, Larter and Lucchitta glaciers. The coloured symbols denote the glacier whose history corresponds to exposure-age data from each sample site (orange circles: Pine Island Glacier; purple squares: Larter Glacier; green diamonds: Lucchitta Glacier); bold symbol outlines and placenames indicate study sites from which the new data were collected. The dashed lines indicate location of main ice divides. Ice surface velocities (blue scale, 0–4218 m yr^−1^)^79^ are overlain on Landsat Image Mosaic of Antarctica (LIMA)^80^ in (**a**) and a Landsat-8 Image acquired on 13 March 2022, courtesy of the U.S. Geological Survey, in (**b**). In both panels, the grounding line (2022; dark blue line) is from ref. ^81^ and the coastline is from ref. ^82^.

This paper presents an integrated data-model comparison of the Holocene deglacial behaviour of the Pine Island Glacier (PIG) system, situated in the Amundsen Sea sector of West Antarctica (Fig. 1a). To link grounding-line dynamics with ice-sheet thinning characteristics, we compare numerical ice-sheet model simulations of the last deglaciation with cosmogenic-nuclide exposure-age data obtained from rock samples collected from the Hudson Mountains, adjacent to PIG (Fig. 1b), during field campaigns spanning the last two decades.

Ever since the Amundsen Sea sector was described as the “weak underbelly of the West Antarctic Ice Sheet” nearly fifty years ago^1^, scientists have regarded PIG (Fig. 1; one of the West Antarctic Ice Sheet’s major outlet glaciers) as vulnerable to runaway retreat. PIG drains an area of 175,000 km^2^^2^. Together with the neighbouring Thwaites Glacier, it has been thinning, retreating and accelerating since the late 1990s^3–5^, driven primarily by the incursion of warm ocean water beneath its ice shelf – the floating extension of the glacier^6,7^.

PIG is currently the largest contributor to global sea-level rise from Antarctica^8^. Modelling suggests its ongoing thinning and retreat will continue in future decades^9,10^ and may be enhanced by anthropogenic ocean forcing^11^. However, the rapid pace of thinning and retreat of PIG observed over the past three decades is not unprecedented. Geological evidence from both marine and terrestrial archives suggests that PIG has retreated and thinned since the Last Glacial Maximum (LGM; ~20 ka), with particularly pronounced changes during the Early and Mid Holocene. Radiocarbon dating of foraminifera in marine sediments offshore in Pine Island Bay showed that the grounding line of PIG had retreated across the continental shelf to reach a location within 120 km of its present position by ~9.2 ka^12,13^ and cosmogenic-nuclide dating of glacial deposits on mountain peaks adjacent to PIG revealed a very rapid phase of thinning at 8–7 ka, during which time the ice sheet thinned by at least 700 m to within 100 m of its current elevation^14,15^.

In an earlier study, Johnson et al.^14^ speculated that such changes were the result of a reduction in buttressing by Pine Island Ice Shelf (PIIS; Fig. 1b), which is thought to have been present in Pine Island Bay until at least 7.5 ka^16^. A similar mechanistic link has been demonstrated elsewhere in Antarctica over a range of scales spanning the modern observational period to the geological past. For example, observational studies before and after the Larsen-B Ice Shelf collapse on the Antarctic Peninsula in 2002 showed that loss of ice-shelf buttressing can trigger subsequent flow acceleration, grounding-line retreat and thinning^17–19^. Similarly, data-model comparisons of Holocene behaviour of East Antarctic Ice Sheet glaciers suggest that reduced buttressing played a role in rapid upstream thinning^20,21^. Numerical ice-sheet modelling of the response of upstream ice to changes at the grounding line also confirms that marine-based ice sheets such as the West Antarctic Ice Sheet (WAIS) are particularly sensitive to changes in ice-shelf buttressing^22^. Several processes can result in reduced buttressing, for example, ocean-induced melting, fracturing, damage, and unpinning from bedrock highs. Retreat, thinning and unpinning can also be driven by rising sea level, as occurred during the demise of the Northern Hemisphere ice sheets following the last glaciation^23^.

To test the earlier speculation and investigate a possible link between weakening of ice-shelf buttressing and upstream thinning in the Holocene (the last 11.7 ka), we compare new ice-sheet thinning histories derived from exposure dating of glacial deposits adjacent to PIG’s former tributaries, Larter and Lucchitta glaciers (Fig. 1b), with existing exposure-age data from adjacent to PIG itself. The new data not only expand the dataset to provide deglacial histories for the tributary glaciers but also substantially increase the density of exposure ages at higher elevations above the modern ice margin, providing new constraint on the maximum LGM ice-sheet thickness.

Both Larter and Lucchitta glaciers presently flow into a northern segment of PIIS that became separated from the rest of PIIS by retreat of the calving front of PIG in 2015^24^. It is not known if this northern ice shelf thinned or collapsed during the Holocene when upstream thinning was taking place. However, from 1996 to 2016 it lost extent and thinned in response to ocean warming^25^, raising the possibility that ongoing warming could further reduce buttressing and cause Larter and Lucchitta glaciers to thin dramatically in the coming decades. Determining how the relative pace and timing of thinning of these tributaries—both individually and collectively—varied in the past would shed light on the mechanisms that drive surface elevation changes in the PIG system.

We present a spatially and temporally detailed thinning history, comprising 82 cosmogenic nuclide (^10^Be) exposure-age data—including 41 new ages and 41 previously-published ages (see Methods)—from erratic cobbles and boulders at 14 nunataks in the Hudson Mountains, a mountain range situated adjacent to PIG and dissected by the two tributaries (Fig. 1b). These data enable us both to track the relative rate and timing of thinning of PIG and its tributaries in the Holocene and to determine the associated impact on ice thickness at individual nunatak sites along a transect extending from near the present grounding line to 75 km upstream^26^. Since the precision of exposure dating is such that it cannot yet reliably capture decadal or centennial-scale changes in ice thickness^27^, the geological data presented here reflect broad patterns of change over millennial timescales and we cannot rule out a different pattern across the PIG system on shorter timescales. Determining broad patterns of millennial-scale change during the Holocene—when Antarctic climate was similar to, or warmer than, today^28^—is nonetheless valuable for constraining the range of ice-sheet behaviours that are possible in the warmer-than-present climate anticipated in coming centuries. Varying temporal thinning and retreat responses of neighbouring glaciers has been detected elsewhere in Antarctica, for example in coastal Dronning Maud Land, where glacier behaviour was broadly synchronous over a few millennia during the Mid Holocene^29^ but asynchronous over the last few decades^30^.

Here, using a high-resolution ice-sheet model that reconstructs ice-sheet evolution on geological timescales, we explore the regional conditions that influenced the observed pattern of thinning of PIG and its tributaries. Models are driven by time-evolving ocean and climate forcings through the last deglaciation. In the Hudson Mountains, however, bed elevation, ice thickness, and ice velocity can vary at much smaller spatial scales than can be represented in coarse-resolution (typically 20 km or higher) continental-scale ice-sheet models; in fact, Larter and Lucchitta glaciers' trunks are not even resolved at these spatial scales. We therefore downscale continental-scale ice-sheet model simulations, performing ‘nested’ modelling^31^ over two targeted domains: the entire Amundsen Sea embayment as a 5 km-grid-resolution domain, to capture fine-scale grounding-line retreat patterns; and Pine Island, Larter and Lucchitta glaciers as a 2 km-grid-resolution domain, to reconstruct high spatial-resolution thinning patterns (see Methods). These nested model simulations better resolve ice-sheet thinning and complex flow patterns across high-relief topography, enabling high-resolution data-model comparison. We use them here to reconstruct the grounding-line backsteps associated with pulses in thinning detected at our sampling sites and thereby attribute thinning events to regional deglacial characteristics. In turn, this allows us to substantiate the previous speculations about the mechanism of rapid onshore thinning that occurred approximately 8–7 ka. We also consider the implications of our findings for future sensitivity of the PIG system to climate change.

## Results

### Exposure-age data – Holocene ice-sheet thinning

**Fig. 2:**
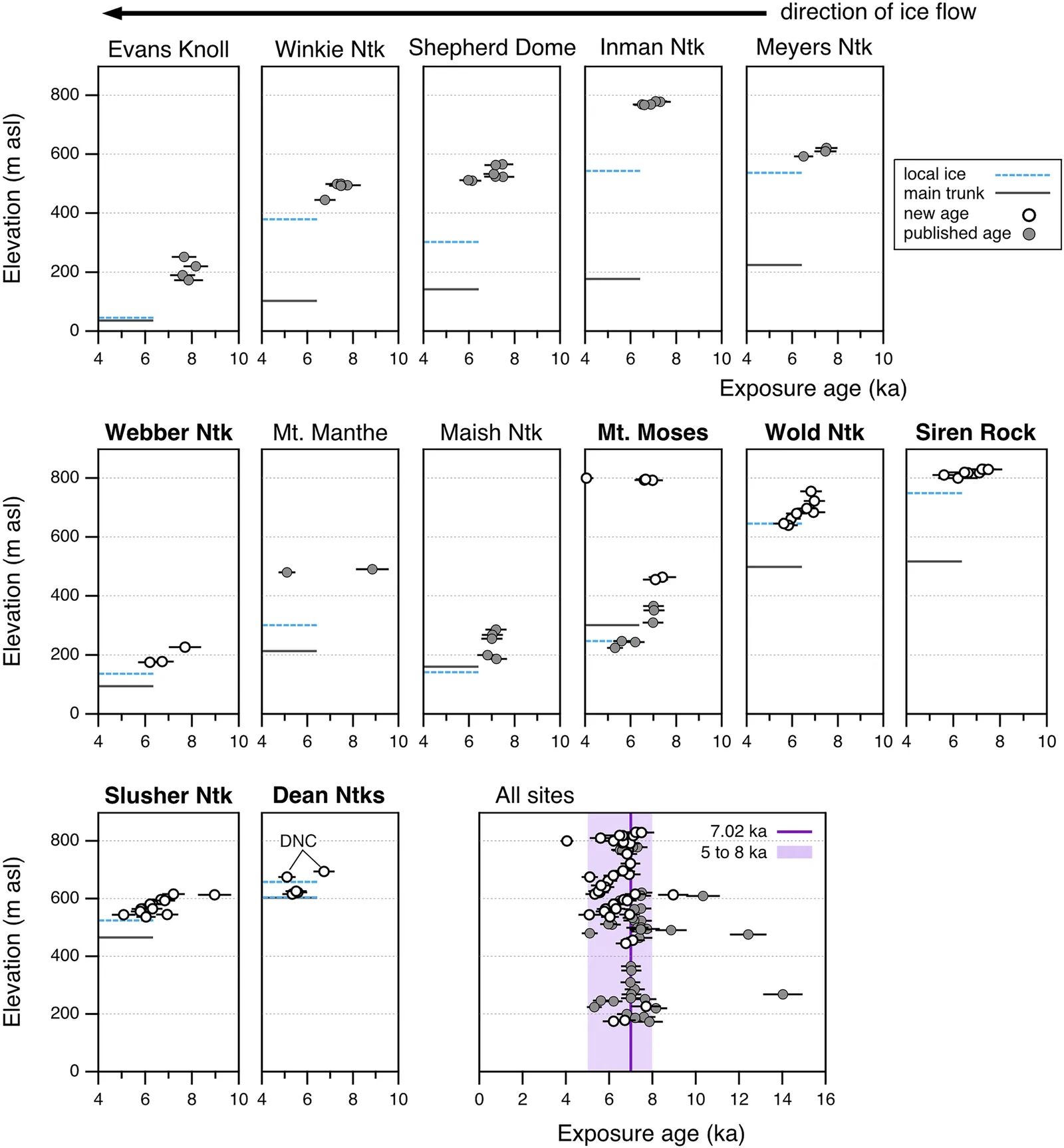
Exposure ages versus elevation for each nunatak, shown along each of the three glacier flowlines. Top row: Pine Island Glacier; middle row: Larter Glacier; bottom row: Lucchitta Glacier. Error bars indicate 1σ external uncertainties. Sites with new data are indicated with titles in bold font and the new data points are represented by white symbols (see legend). At Dean Nunataks, “DNC” refers to samples from the crater; the other symbols relate to samples from the knoll (see Methods). Dashed blue lines indicate elevation of the local modern ice-sheet surface adjacent to each nunatak and horizontal solid black lines indicate elevation relative to the main glacier trunk (see Supplementary Information for how these elevations were determined). The exposure ages cluster around 7.0 ka (mode 7.02 ka; Supplementary Fig. 1). Note: Three samples at Mount Moses were collected from erratic boulders situated below the modern ice surface next to a windscoop (see ref. ^14^); they were not sampled subglacially.

Exposure histories derived from ^10^Be exposure age versus elevation profiles from 14 of the Hudson Mountains nunataks are shown in Fig. 2. The 82 exposure ages range from 14.0 ± 0.9 to 4.0 ± 0.3 ka, with the vast majority (all but six) in the range 8.2 ± 0.5 ka to 5.1 ± 0.5 (Supplementary Data 1), with clustering around 7.0 ka (Supplementary Fig. 1). None of the erratics sampled yielded exposure ages older than 14.0 ka and only three are older than 10 ka (MEY-102, UNN-106 and JF-03, with ages 10.3, 12.4 and 14.0 ka, respectively; Fig. 2), implying that none have retained significant inherited ^10^Be from exposure prior to the last glacial period. At all sites, the exposure ages get younger downhill. Together, they show lowering of the ice-sheet surface by up to 700 m between 8 and 5 ka. We interpret this as thinning of the three glaciers flowing through the Hudson Mountains: Lucchitta Glacier, Larter Glacier and Pine Island Glacier^26^.

Samples from erratic cobbles collected from the summits of the highest exposed peaks in the Hudson Mountains (Siren Rock, 830 m asl; Mount Moses, 800 m asl; Inman Nunatak, 779 m asl), which today rise >500 m above the modern ice sheet surface, yielded ^10^Be exposure ages in the range 7.5 ± 0.5 ka to 4.0 ± 0.3 (Supplementary Data 1). This finding supports inferences from glacial-geomorphological observations^26^ suggesting that all the Hudson Mountains nunataks were completely covered by the ice sheet during the LGM (19–26.5 ka^32^) and implies that it is not possible to determine the maximum LGM ice sheet thickness from any sites there because none were exposed above the ice-sheet surface at that time.

Similarly, exposure ages of Late Holocene age (<4.2 ka^33^) are almost entirely absent in our dataset (Fig. 2), including at nunataks where sampling extended down to the modern ice surface. Only one sample, MOS-104, yielded such a young age (4.0 ± 0.3 ka). It was collected from a col close to the summit of Mount Moses, 500 m above the present ice-sheet surface. Samples from three further erratics at the same elevation (MOS-101, MOS-102 and MOS-103) have ages that overlap within error (7.0 ± 0.5, 6.6 ± 0.4 and 6.7 ± 0.4 ka, respectively), suggesting that the 4.0 ka age is anomalous and not a reliable deglaciation age; other than perhaps the presence of prolonged local snow cover, we have no explanation for why this sample has such a young age. Exposure ages collected from samples close to (within 60 m) of the modern ice surface at several of the nunataks (Winkie, Meyers and Maish nunataks, Dean Nunataks Knoll and Mount Moses; 7.2 ± 0.5 to 5.3 ± 0.4 ka) imply that the ice-sheet surface had reached its modern elevation by ~5 ka across the Hudson Mountains.

Glacial-geomorphological observations imply that the exposure ages can be interpreted as reflecting regional ice-sheet thinning rather than retreat of a local ice cap. Specifically, erratics are of exotic lithology^26^ suggesting a non-local source^34^, there are no moraines present that would indicate locally fed glaciers^26^, and striated bedrock was not observed on nunatak summits^26^. Furthermore, the rare striations that exist on bedrock surfaces at lower elevations are not oriented radially outward from the summits as would be expected for retreat of a local ice cap^26^.

The timing of ice thinning determined from the exposure-age dating is broadly synchronous across the Hudson Mountains (Fig. 2). However, the very high spatial density of the dataset—with data from nunataks close to the grounding line as well as at several intervals up to 75 km upstream along the three glaciers—allows us to detect heterogeneity in past ice-sheet thinning behaviour that is often not identifiable in exposure-age datasets that are smaller or restricted to just a few nunataks. We observe the following key features. Firstly, the average rate of thinning (reported as the average over tens to hundreds of metres of thinning; Supplementary Table 1) was not identical between sites. Secondly, at some sites, there is evidence for changes in thinning rate through time. For example, at Mount Moses, initially rapid thinning was followed by a period of slower thinning as the ice sheet neared its modern surface elevation. At Maish Nunatak and Evans Knoll, only rapid thinning is observed, but at those sites, absence of data from within 34 and 144 m of the modern ice sheet surface, respectively, means we cannot rule out a later slower phase. In contrast, thinning was steady (and, on average, slower) at other sites such as Slusher Nunatak and Siren Rock. Lastly, at a few sites (specifically Meyers Nunatak, Webber Nunatak, Dean Nunataks), there is insufficient density of data or sampled elevation range to permit determination of the pattern and/or rate of thinning.

### Model simulations – regional context for ice-sheet thinning

**Fig. 3:**
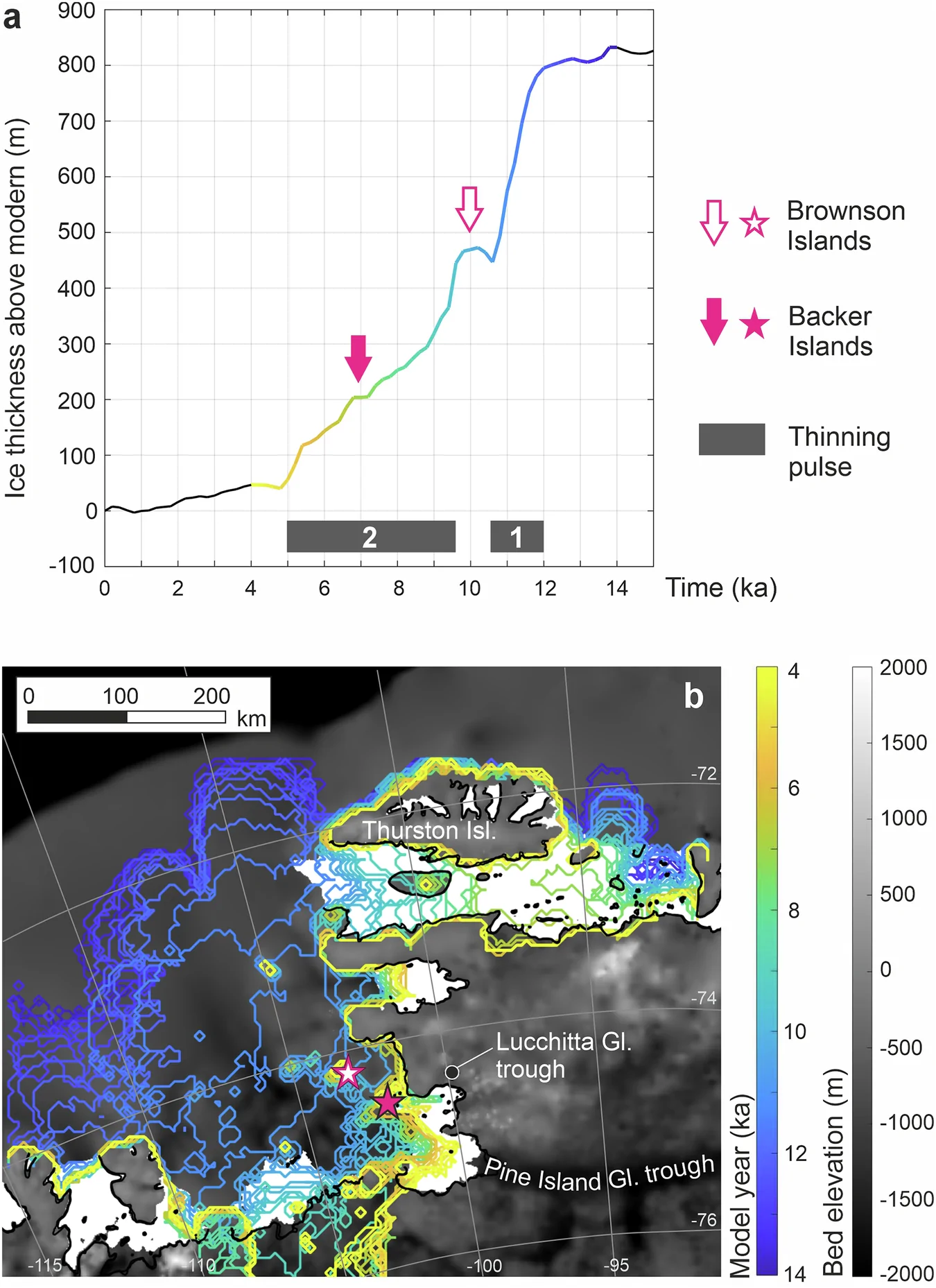
Modelled deglaciation of the Lucchitta Glacier trough and corresponding model grounding-line positions. **a** Modelled thinning profile of Lucchitta Glacier. Colours correspond with model years, as in (**b**). Arrows indicate two pauses in thinning mentioned in the text (filled arrow indicates pause that corresponds with pinning on Backer Islands, and open arrow corresponds with pinning on Brownson Islands; see (**b**) for locations). **b** Map showing model grounding-line positions from 14–4 model ka, with modern ice shelves presented in white and modern grounding line as a black line. Note: This figure shows deglaciation of Lucchitta Glacier trough; in the model, Lucchitta and Larter glaciers both experience the same history of marine forcing, and their modelled flowlines likewise converge (see also Fig. 5).

The output from our high-resolution model provides a physics-based and internally consistent framework that allows us to link thinning patterns at the Hudson Mountains nunatak sites to regional deglacial behaviour. Using Lucchitta Glacier as an example, Fig. 3 shows how modelled ice thickness changes as the marine grounding line retreats across the continental shelf of the Amundsen Sea embayment. It reveals an overall stepwise thinning trajectory—pulses of fast thinning interspersed with short pauses in thinning—associated with discrete grounding-line backstepping.

**Fig. 4:**
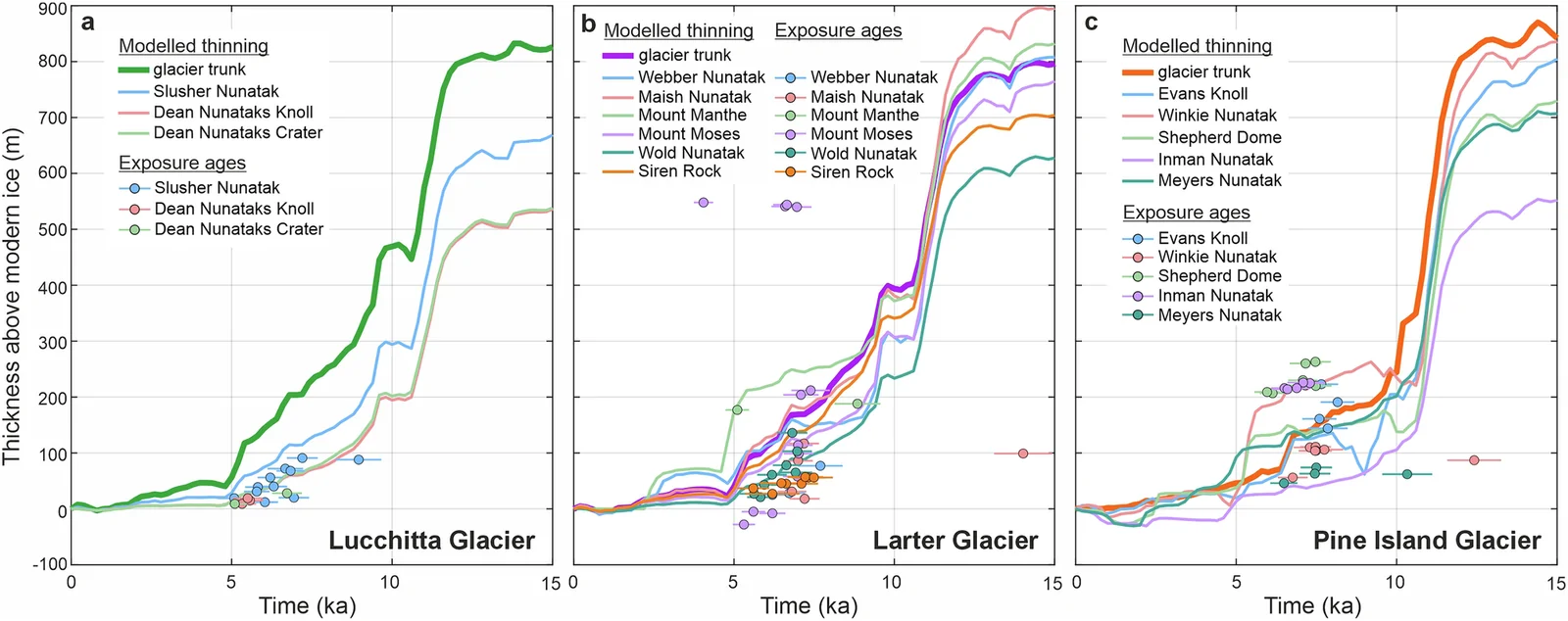
Comparison of exposure-age data with modelled thinning profiles for Pine Island Glacier and its tributaries through the Holocene. Each panel shows the averaged modelled ice-sheet thinning profile (15 ka to present; six points each) for the centreline of each glacier (thick line) and at nunatak sites (thin lines), alongside exposure-age data from the adjacent nunataks (coloured circles with error bars indicating 1σ external uncertainties; Supplementary Data 1). **a** Lucchitta Glacier trough and exposure ages from Dean Nunataks and Slusher Nunatak. **b** Larter Glacier trough and exposure ages from nunataks on both its northern margin (Maish Nunatak, Mount Moses and Siren Rock) and southern margin (Webber Nunatak, Mount Manthe and Wold Nunatak). Note: Three samples (from Mount Moses) are located below the modern ice elevation next to a windscoop (see ref. ^14^). **c** Pine Island Glacier trough and exposure ages from Evans Knoll, Winkie Nunatak, Shepherd Dome, Inman Nunatak and Meyers Nunatak. In the model, ice elevation is represented as thickness above modern (time = 0). The spatial evolution of ice thinning at these sites is shown in Supplementary Movie 1.

A broadly similar pattern and timing of ice-sheet elevation change is generated by the model along the trunks of PIG and Larter Glacier (Fig. 4); thus, to a first order, all three glaciers in the PIG system appear to have thinned in concert. The first pulse of modelled thinning is rapid (Pulse 1, average rate of 0.24–0.33 m yr^−1^; Supplementary Table 1) and is associated with grounding-line recession across the outer- and mid-continental shelf (Fig. 3b; initiated at ~12 ka in this model simulation). A subsequent pause in thinning (here, initiated at ~10.6 ka) corresponds with the model grounding line reaching the Brownson Islands on the inner continental shelf (Fig. 1a). At Lucchitta Glacier, this hiatus is accompanied by a short-lived (~1000-year) increase in model ice thickness (Fig. 3a) while at PIG and Larter Glacier the hiatus is less pronounced, resulting in a pause in thinning but no associated increase in ice thickness. Thinning after the pause continues at a rapid pace, but slower than during Pulse 1 (Pulse 2, average rate of 0.03–0.07 m yr^−1^; Supplementary Table 1). Another short pause then occurs (at 7 ka; Fig. 3a) as the ice sheet becomes briefly pinned on the Backer Islands in Pine Island Bay (Fig. 3b). During the final phase of grounding-line retreat (reaching modern limits by ~5 model ka in this simulation; Fig. 3b), thinning is markedly slower.

Although the modelled thinning pattern through the Holocene is broadly synchronous across the Hudson Mountains, finer-scale variations are apparent at some nunatak sites adjacent to PIG and Larter Glacier after 10 model ka (the ice sheet stays thicker for longer at Mount Manthe, Winkie Nunatak and Shepherd Dome than at other sites, for example; Fig. 4 and Supplementary Movie 1). These variations are likely related to local ice-flow patterns around nunataks, which produce features such as windscoops and local thickening^35^. The presence of these variations only in the later stages of deglaciation suggests that bedrock topography has an increasing influence on the pattern of thinning as the ice-sheet surface lowers through the last 200–300 m above present elevation.

We estimate that the overall regional (across whole PIG catchment) reduction in ice-sheet volume associated with thinning/retreat Pulses 1 and 2 contributed 0.0889 m to eustatic sea-level change in 7000 years. The model indicates a stepwise pattern in the trajectory of both grounding-line retreat and thinning of grounded ice at the inland nunatak sites, providing regional context for the measured thinning rates.

### Data-model comparison – rates and phases of Holocene ice-sheet change

A common challenge when comparing modelled deglacial histories with geological datasets is that geological observations, such as thinning histories derived from exposure dating, are often discontinuous and are unlikely to capture the full deglacial history. It can also be difficult to determine the exact relationship between sample and model elevations, and thus which phase(s) of a site’s deglacial history are recorded by the exposure-age data.

Figure 4 shows the relationship between modelled and observed thinning at sites adjacent to PIG, Larter and Lucchitta glaciers. Most of the empirical data overlap with the modelled thinning profiles and correspond with modelled thinning Pulse 2. However, some data-model mismatches are still evident (cf. ref. ^36^) and model representations of finer details of thinning, particularly in the latter phases of deglaciation, are not reflected in the empirical data (particularly at nunataks adjacent to Larter Glacier and PIG; Fig. 4b, c). The data-model comparison further suggests that the exposure-age data may not be capturing a possible earlier faster phase of thinning (modelled thinning Pulse 1), even though we sampled the summits of the highest nunataks in the Hudson Mountains. The only samples that might have been high enough to constrain Pulse 1 are those from the uppermost slopes of Mount Moses (Fig. 4b), but unlike the majority of the dataset, their exposure ages appear to record deglaciation 5000 years later than modelled. It thus remains plausible that the modelled LGM ice-sheet thickness is too high. An alternative explanation is that the high-elevation exposure ages reflect later deglaciation of a local ice cap or locally-fed glaciers rather than the main glacier trunks, but this interpretation is not supported by geomorphological evidence (see Results). Notwithstanding these challenges, although discontinuous, the overall thinning pattern provided by our empirical data is broadly consistent with the model simulations, implying that we can use the model to provide physical context for our data-based observation of concerted ice-sheet thinning across the PIG region.

## Discussion

Here we use the relationships between the measured thinning histories and modelling of the marine grounding line to determine possible influences on Holocene surface-elevation changes at PIG and its tributary glaciers.

### Regional drivers of Holocene ice-sheet thinning

**Fig. 5:**
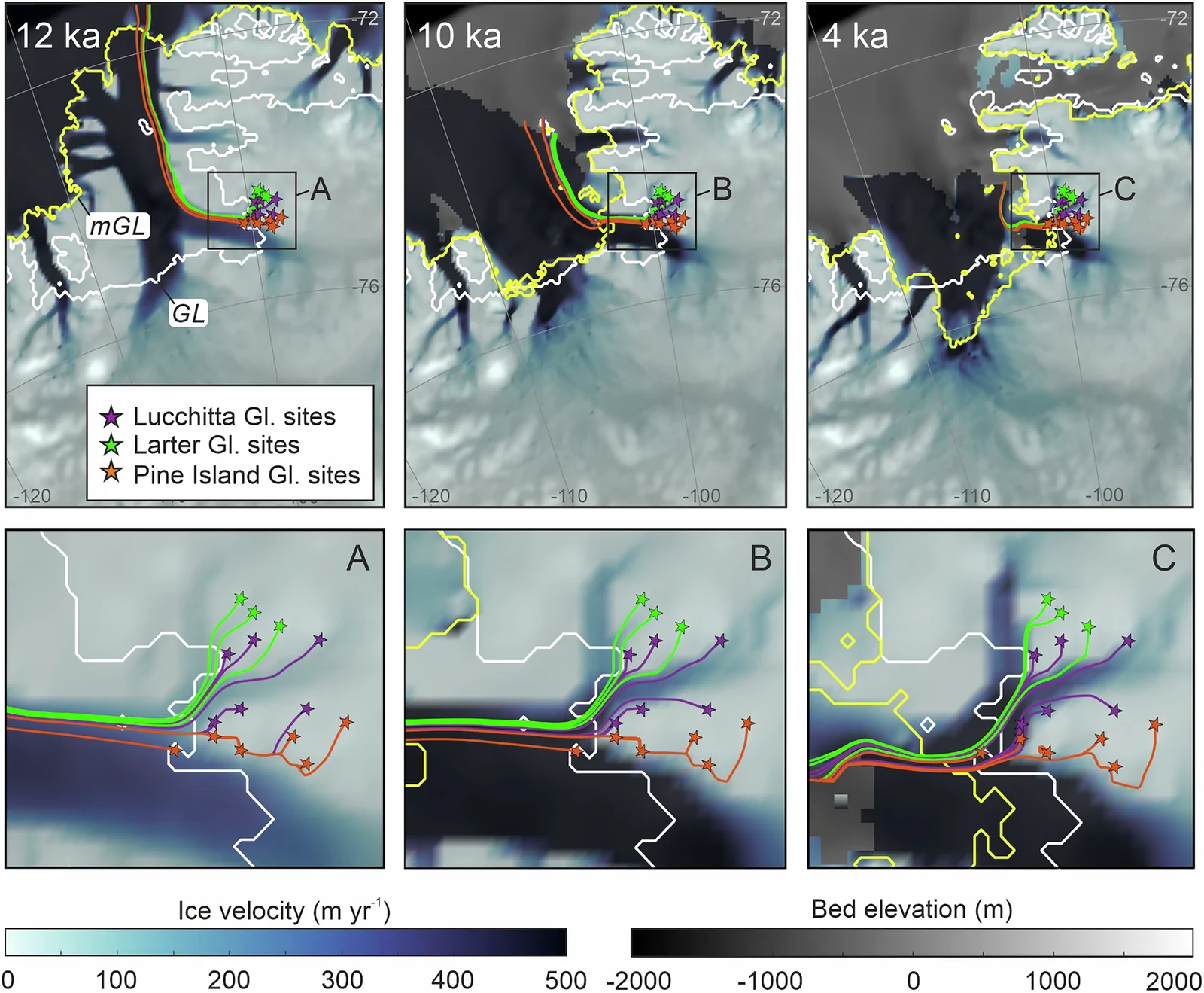
Modelled ice flowlines from sample sites in the Hudson Mountains through the episode of rapid thinning (panels show model snapshots at 12, 10 and 4 ka). At all timesteps, the flowlines in Lucchitta (green), Larter (purple), and Pine Island (orange) glacier troughs converge downstream of the sample sites. **A**–**C** show close-up views of the location of sample sites in the Hudson Mountains (coloured stars) and modelled flowlines (solid lines, corresponding colours) feeding the three glaciers. The modelled (labelled “*mGL*”) and observed (modern; labelled “*GL*”) grounding lines are represented by the yellow and white lines, respectively. In all panels, bed elevation is displayed underneath ice velocity (scale bars below panels).

The relatively short time range covered by the ^10^Be exposure-age dataset (8.2–5.1 ka, with the majority of ages clustered around 7 ka) suggests that the glaciers presently draining the Hudson Mountains (PIG, Larter and Lucchitta) thinned broadly synchronously during a period of less than 3000 years in the Mid Holocene. To understand what drove this concerted behaviour, we examined modelled grounding-line position and ice-flow paths through the main episode of rapid Holocene ice-sheet thinning (Fig. 5). Modelled ice flowlines extending from our sample sites in the Hudson Mountains all converge downstream and follow a similar path across the continental shelf during the deglaciation. Thus, the flow of each glacier is affected throughout the deglaciation by the same downstream dynamic forces from a common marine terminus region. It follows that any changes in buttressing are expected to have affected PIG and its tributaries simultaneously, resulting in a synchronous pattern of thinning across the Hudson Mountains region. This is what we observe in the thinning histories obtained from the glacial erratics. Their shared deglacial history and the similarity in the overall pattern of model thinning (and buttressing – see later discussion) of PIG, Larter, and Lucchitta glaciers therefore suggests that their deglacial evolution was controlled by regional-scale processes and shared boundary conditions, with the minor differences resulting from local bedrock geometry. Such regional-scale processes were likely driven by inflow of warm Circumpolar Deep Water, which was enhanced in the Amundsen Sea sector following the LGM and into the Early Holocene, including immediately prior to the observed thinning of the PIG system^37^. The direct influence of warm Circumpolar Deep Water on Holocene ice-shelf instability and inland ice-sheet thinning has recently been demonstrated with ice-sheet modelling in East Antarctica, where thinning occurred simultaneously with Pulse 2 of the PIG system via a mechanism induced by meltwater release from the retreating WAIS^38^.

### Influence of the seafloor on Holocene ice-sheet retreat and thinning

**Fig. 6:**
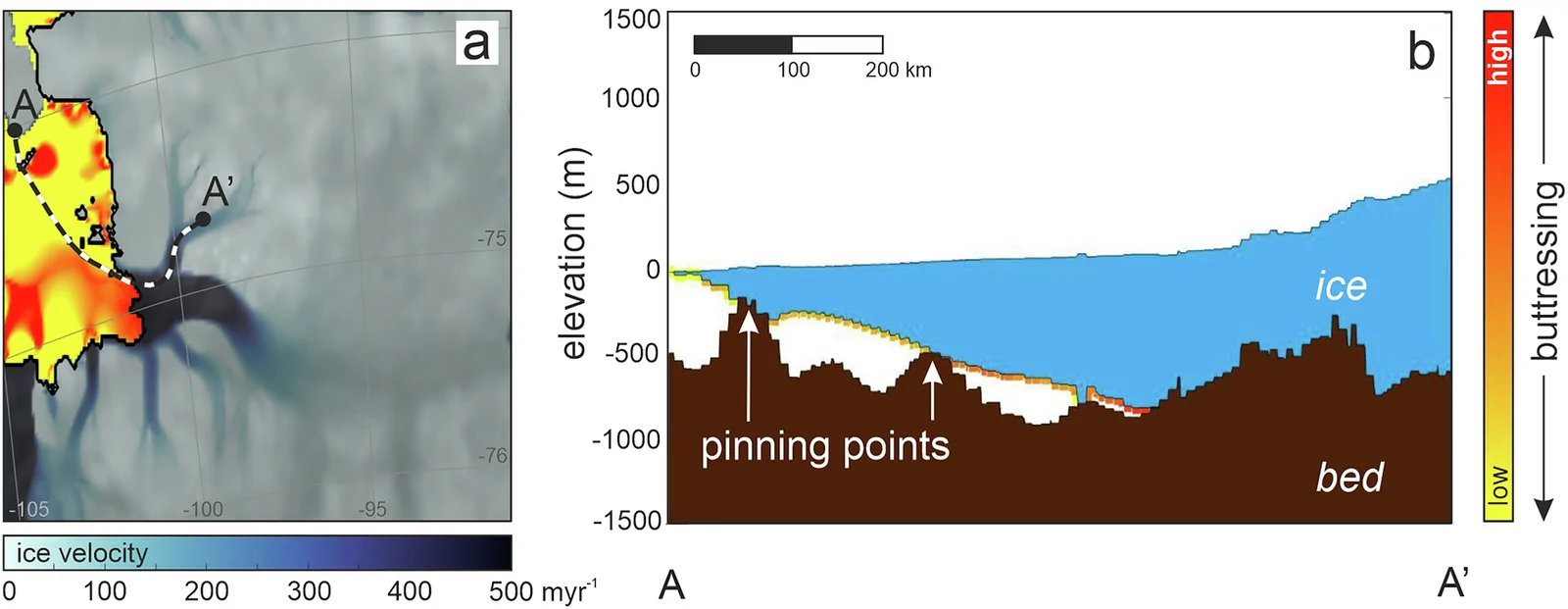
Modelled ice-shelf buttressing of the Pine Island Glacier system. **a** The Pine Island Glacier system at 10.4 model ka, with a flowline (dashed line, A-A’) propagated along the Larter Glacier trough. The ice shelf colour reflects the buttressing factor (see Methods), with yellow representing little to no buttressing, and red indicating a high degree of buttressing. **b** Cross section of the flowline shown in (**a**). The ice sheet and ice shelf are represented in blue, and the bed is represented in dark brown. Two distinct pinning points occur under the floating portion of the ice shelf. The ungrounded portions of the ice-shelf base are coloured according to buttressing factor, indicated by the vertical scale bar (right).

Cross-correlating our geological data and modelled thinning profiles enables exploration of where the grounding line was likely situated during specific phases of Holocene ice-sheet elevation change and how the seafloor topography influenced deglaciation. Figure 6 shows the model grounding-line location and concurrent ice-sheet geometry for a snapshot corresponding with a pause between modelled thinning Pulses 1 and 2 at all three glaciers (see Fig. 4). The flowline intersects with two distinct bedrock topographic highs on the inner continental shelf (Fig. 6a) that are co-located with the presently exposed Brownson and Backer Islands groups (Fig. 3). During the pause, the ice shelf is in contact with these highs, resulting in increased buttressing on their upstream side (Fig. 6b). The model suggests that ice was grounded (‘pinned’) on the bedrock highs for a period long enough to exert back stress (which provides resistance to inland sliding and thinning), impeding the flow of ice downstream through the Hudson Mountains and into the northern PIIS. It links these pinning points to an abrupt change in the rate of upstream thinning along each glacier that lasted up to 1000 model years (Fig. 4) – temporary slowing (PIG; Fig. 4c), stabilisation (Larter; Fig. 4b) or reversal (Lucchitta; Fig. 4a), respectively. The duration of these changes is broadly consistent with the extent of an earlier stillstand recorded by a grounding-zone wedge situated on the middle continental shelf near Burke Island (“GZW5”)^39^ that is estimated to have taken 600–2000 years to form^40^. After this pause, resumption and/or acceleration of modelled thinning is correlated with unpinning from the topographic highs and accompanied by a concomitant increase in the rate of grounding-line retreat.

This model exploration of onshore thinning patterns modulated by marine pinning points can be applied to the geological records of deglaciation of the PIG system. Specifically, the results support a combination of two earlier hypotheses proposed by ref. ^14^ to explain rapid mid-Holocene thinning in the Hudson Mountains: rapid landward migration of the PIG grounding line after decoupling and unpinning from a topographic high, and a reduction in ice-shelf buttressing triggered by the unpinning. The results also highlight the role of seafloor pinning points in driving temporary grounding-line stabilisations that in turn impact inland ice-surface elevation. This process continues to be important for predicting future changes in the Amundsen Sea sector as it has been modelled as a key driver of future retreat of the neighbouring Thwaites Glacier^9^.

### Importance of a holistic approach to understanding drivers of regional deglaciation

Our study demonstrates the benefit of a combined data-model approach for contextualising palaeo records of ice-sheet change derived from geological datasets. Here, rather than interpreting evidence from the data-based thinning records in isolation, we contextualise them using ice-sheet modelling. This holistic approach ensures our interpretations of data-based observations of ice-sheet behaviour have a robust physical basis. For example, relating the pattern and timing of both past inland ice-sheet thinning and grounding-line retreat across the continental shelf along individual glacier flowlines enabled us to i) determine the relationship between ice-sheet elevation changes at intervals up to 75 km upstream as well as at the grounding line, and ii) determine the possible drivers of these changes by linking them to physical processes. We show that the rate of thinning of glacier ice flowing through the Hudson Mountains in the Holocene can be attributed to variations in back stress induced by pinning/unpinning of the retreating ice shelf on seafloor topographic highs in Pine Island Bay. This demonstrates that the topography of the bed in Pine Island Bay, over which the ice sheet slides during retreat, plays a key role in driving upstream ice-sheet behaviour.

Our holistic approach also highlights challenges and nuances associated with correlating data with model simulations. The high spatial and temporal resolution of our exposure-age dataset enabled exploration of the relationship between offshore grounding-line retreat and inland thinning at different times during deglaciation in a single catchment. However, due to the logistical and technical difficulties commonly encountered when working in the remote polar regions, exposure-age datasets from those areas are often geographically sparse and discontinuous (as an example, it took three field campaigns conducted over the course of 14 years to build the detailed Hudson Mountains exposure-age dataset presented here). Thus, they typically provide information covering only snapshots of the last deglaciation, making it challenging to link them either together and/or with other geological records such as those from marine sediments. For example, although we know from the marine record that retreat of grounded ice from the eastern Amundsen Sea embayment was underway by 20.6 ka^13,41^ and reached close to the current configuration by 9.2 ka^12^, our exposure-age data record thinning in the Hudson Mountains over only a comparatively short—and later—period (~8–5 ka). There is thus no temporal or spatial overlap between the two datasets. It follows that they record different deglacial events and accordingly we should not routinely assume that an episode of rapid onshore thinning must correspond with the period of greatest marine grounding-line backstepping. Furthermore, a data gap covering the last ~5000 years of thinning history of the PIG system remains, leaving open the possibility that PIG and its tributaries may have thinned below the modern surface and rethickened during this period (as was detected 300 km west near Pope Glacier [Fig. 1a] via recovery and cosmogenic nuclide analysis of subglacial bedrock^42^). Nested ice-sheet modelling can help overcome such data limitations by interpolating to simulate a thinning and retreat history that is consistent with all available geological datasets and covers the full deglacial period, not just snapshots in time.

### Implications for future ice-mass loss from Pine Island Glacier

Finally, our synthesis of new and existing exposure-age data from the Hudson Mountains provides evidence that the Hudson Mountains were covered by the Antarctic ice sheet during the Early Holocene (and probably also at the LGM), concurring with earlier model predictions of ice-sheet thickness in the Amundsen Sea embayment during the past 20,000 years^36^). Rapid regional deflation of the PIG system followed into the Mid Holocene. The later phase of this deflation is reflected in our geological data and physically-based model as broadly synchronous thinning across the Hudson Mountains (albeit with finer-scale variations in local ice-surface elevation changes around nunataks; see earlier discussion). The concerted nature of this thinning across all three glaciers, including Larter and Lucchitta glaciers that currently feed the northern PIIS, implies that the glaciers experienced similar buttressing throughout the deglaciation. The associated reduction in ice-sheet volume across the PIG catchment would have contributed up to 90 mm to eustatic sea level rise over 7000 years in the Early to Mid Holocene and may have been temporarily stabilised by episodic ice-shelf pinning on seafloor highs on the inner shelf of Pine Island Bay.

The results of our study demonstrate that, if a reduction in buttressing simultaneously affects several tributaries that feed a major outlet glacier in a marine-based ice sheet such as WAIS (as occurred in the Mid Holocene in the PIG system), there is potential for rapid regional ice-mass loss. Since they impact pinning, the topography and properties of the bed influence the rate and style of both retreat and associated inland thinning. Although knowledge of the bed topography beneath the northern PIIS is very limited (only poorly constrained airborne gravimetry observations exist^43–45^), it is likely that the rugged crystalline bedrock interspersed with occasional pockets of sediment that dominates the seafloor of inner Pine Island Bay^46,47^ (Supplementary Fig. 2) extends beneath it. Thus, additional pinning points that might help stabilise future retreat are likely present. Several ice rumples measuring a few kilometres in length are visible in the surface of the northern PIIS (Supplementary Fig. 2). Such prominent surface features indicate locations where the ice shelf is currently pinned on the underlying bed^48^, providing buttressing (to upstream flow) that would be reduced or lost if unpinning were to occur in future. If the PIG system continues to be subjected to incursion of warm Circumpolar Deep Water beneath its ice shelves^16^—perhaps in future amplified by anthropogenic-induced forcing trends^11,49^—grounding-line stabilisation via pinning on seafloor highs would likely be only short-lived, and further retreat and loss of ice would not pause for long. The consequence could be another episode of rapid widespread thinning across the PIG catchment, with an associated rise in sea level. Although glacial-isostatic uplift resulting from continuing ice-mass loss^50^ could provide a counter-effect^51^, coupled ice-ocean modelling of the PIG system^9^ (Supplementary Fig. 3) suggests that unpinning and associated regional ice-sheet deflation could occur within the next century, possibly even without further anthropogenic forcing.

## Methods

### Field sampling

In this paper, we present new cosmogenic-nuclide analyses of 41 erratic cobbles and boulders from the Hudson Mountains and compile these with all other published exposure-age data from the area (the complete dataset comprises a total of 82 exposure ages; Supplementary Data 1). Forty-one previously-published exposure ages are from granitoid erratic cobbles and boulders collected over three field seasons (2006, 2010, 2019-20; by J.S.J. and James Smith, British Antarctic Survey) and are reported in refs. ^14,15,52^. Site descriptions and field sampling methods for the 41 new erratics are described in detail in ref. ^26^; we provide a short summary in the paragraph below.

Samples from the new erratics were collected by J. S. J. (under UK Foreign & Commonwealth Office section 6 permit #BAS-S6-2019/10) during the 2019-20 austral summer season, as part of a field campaign supported by British Antarctic Survey Operations Team for the International Thwaites Glacier Collaboration ‘Geological History Constraints’ project. Of the 17 Hudson Mountains peaks, 15 were visited and exposure ages were obtained from 14 of those; Velie Nunatak and Koehler Nunatak (Fig. 1b) were not accessed due to both time constraints and challenging terrain (including extensive crevassing between Slusher and Velie nunataks), and cobbles at World’s End Bluff were not analysed because their depositional history was uncertain (possibly weathered out of the underlying bedrock rather than glacially-transported; see ref. ^26^). Erratics of exotic lithology (i.e. not derived from locally outcropping bedrock) perched on horizontal or gently sloping bare/scoured bedrock (entirely free of till, sediment, soil or snow) were preferentially chosen for sampling to minimise the chance of periodic snow cover or post-depositional movement associated with periglacial activity or mass wasting, as these would affect the interpretation of cosmogenic-nuclide measurements. The position (latitude, longitude, elevation) of each erratic was measured with a Javad Triumph II GPS receiver and the heights above ellipsoid corrected to orthometric heights (height above geoid EGM08) using Precise Point Positioning software. At all sites, the degree and direction of topographic shielding of each erratic were measured using a compass clinometer and recorded along with the geomorphological setting. Sample information required for exposure-age calculation (latitude, longitude, elevation, topographic shielding correction factor, and sample thickness) is provided in Supplementary Data 2.

### Cosmogenic-nuclide analysis

As described above, analytical data and methods for the existing exposure ages are reported in refs. ^14,15,52^. For the new analyses, analytical data and exposure ages (including ^10^Be/^9^Be ratios and ^10^Be concentration) are provided in Supplementary Data 3, procedural blanks are provided in Supplementary Data 4 and information formatted for online exposure-age calculator input are provided in Supplementary Data 5. We describe the laboratory and analytical methods used to determine the new exposure ages below.

We prepared 41 purified quartz samples for cosmogenic-nuclide analysis in the CosmIC Laboratory, Imperial College London. We applied typical quartz isolation procedures (e.g. ref. ^53^), including crushing, milling, sieving, magnetic separation, froth flotation^54^, HCl leaching, and HF-HNO_3_ etching. Quartz purity was tested using inductively-coupled plasma optical-emission spectrometry (ICP-OES). We performed isotope-dilution chemistry for ^10^Be analysis using the methods described by ref. ^55^. For each sample, as well as three repeat measurements, we dissolved between 3.8 and 28.7 g of pure quartz (Supplementary Data 3) and approximately 338 mg of low-background ^9^Be carrier solution (775.23 µg Be g^−1^ and ^10^Be/^9^Be = 2.6 ×10^−16^ ± 6.9 ×10^−17^; Supplementary Data 3) using concentrated hydrofluoric acid (HF) and nitric acid (HNO_3_). We also prepared three ~0.5 g aliquots of quartz of the laboratory intercomparison material CRONUS-A^56^ (see below).

We measured ^10^Be/^9^Be ratios at the Centre for Accelerator Science, Australian Nuclear Science and Technology Organisation (ANSTO) using procedures described in refs. ^57,58^. The ^10^Be/^9^Be measurements are normalised to the KN-5-3 standard with an assumed ratio of 6.320 ×10^−12^ (t_1/2_ = 1.36 Ma^59^; Supplementary Data 3). Two secondary standards were run as unknowns to confirm the linearity, accuracy, and precision of the measurements. One procedural blank (carrier-only) was processed for every 11 samples, and each batch of samples was assigned a batch number (see Supplementary Data 3–5). The procedural blank used to correct the ^10^Be concentration of each sample is dependent on the period during which it was processed in the CosmIC Laboratory (see Supplementary Data 4). Beryllium was extracted from samples in batch 1 at the same time as the samples presented in refs. ^15,42,60^. Therefore, the blank value used to correct the ^10^Be concentrations in batch 1 is the same as that used to correct ^10^Be concentrations in the aforementioned studies (the mean and standard deviation of 12 batches of samples, ^10^Be/^9^Be = 1.73 ×10^−15^ ± 4.82 ×10^−16^, 30287 ± 8456 ^10^Be atoms; Supplementary Data 4). Samples in batch 2 were processed in the year following those in batch 1 and are corrected with a different blank value (the mean and standard deviation of two process blanks, BLK300822 and BLK050922, ^10^Be/^9^Be = 2.71 ×10^−15^ ± 3.61 ×10^−16^, 47410 ± 6255 ^10^Be atoms; Supplementary Data 4). Samples in batches 3–5 were processed later in the same year as those in batch 2 and the procedural blanks for batches 3–5 exhibit a greater degree of scatter than those in previous batches. We therefore used a batch-specific blank value to correct the ^10^Be concentrations of samples in batch 3 (BLK120922, ^10^Be/^9^Be = 2.13 ×10^−15^ ± 3.41 ×10^−16^, 37088 ± 5939 ^10^Be atoms), batch 4 (BLK260922, ^10^Be/^9^Be = 1.86 ×10^−15^ ± 3.88 ×10^−16^, 32550 ± 6786 ^10^Be atoms), and batch 5 (BLK041022, ^10^Be/^9^Be = 4.50 ×10^−15^ ± 5.31 ×10^−16^, 78589 ± 9262 ^10^Be atoms; Supplementary Data 4). In all cases, uncertainties in the sample and blank values are propagated in quadrature.

To verify accuracy and reproducibility, we also measured the ^10^Be concentration of three field replicates (samples SIR-104R, WOL-104R, and MOS-105R; Supplementary Data 3) and three aliquots of the laboratory-intercomparison material CRONUS-A^56^ (Supplementary Data 3). The ^10^Be concentrations of samples SIR-104R, WOL-104R, and MOS-105R differ from their accompanying measurements by 6.1, 3.9, and 6.3 %, respectively, which are within 1.65σ (90 % confidence) uncertainties. We therefore use the average and standard deviation of the two ^10^Be concentrations for each sample in the exposure-age calculations described below. The three aliquots of CRONUS-A yield ^10^Be concentrations of 3.43 ×10^7^ ± 8.38 ×10^5^, 3.60 ×10^7^ ± 6.99 ×10^5^, and 3.50 ×10^7^ ± 6.73 ×10^5^ atoms g^−1^ which are only ~0.4, 5.3, and 2.4 % different from, and therefore consistent with, the consensus value of 3.42 ± 0.1 ×10^7^ (1σ) atoms g^−1^ of ref. ^56^ within 2σ uncertainties.

### Exposure-age calculations

Exposure ages (both new and existing) were calculated using version 3 of the online exposure-age calculators formerly known as the CRONUS Earth online calculators (http://hess.ess.washington.edu)^61^ with the “primary” production rate calibration dataset^62^. The online calculators use the “LSDn” production rate scaling method for neutrons, protons, and muons following ref. ^63^ and summarised in ref. ^64^. For all samples, we used a sample density of 2.7 g cm^−3^ and assumed no erosion and a single period of exposure. In Supplementary Data 3, we also include exposure ages calculated using the “St” production rate scaling method^65^ to aid comparison with other studies and to assess the impact of the choice of scaling method on the resulting exposure ages. For the new exposure ages, use of the St scaling method produces ages on average ~400 years (or 5.6 %) older than those using the LSDn method (see Supplementary Data 3). Thus, choice of scaling method does not alter our interpretations. We do not use a glacial-isostatic adjustment (GIA) correction to calculate the exposure ages. Whilst use of a GIA correction would make the calculated ages slightly older, this offset may be counteracted by incorporating the effect of atmospheric redistribution on past production rates.

### Ice-sheet modelling

We leverage existing model frameworks to provide physical context for the measured terrestrial thinning histories. For this, we use a 3-D ice-sheet model (PSU-ISM) that has been widely applied to simulate ice-sheet evolution on geological timescales^66,67^. We perform three different high-resolution nested model simulations with varied parameters to provide different physical realisations of the last ice-sheet deglaciation in support of our analysis of local thinning patterns and their regional footprint, but do not attempt ad-hoc parameter tuning to precisely reconstruct the retreat chronology with our simulations. The 3-D velocity field (x and y components) is computed for each discrete element of the model, at every timestep of the simulations.

The PSU-ISM model iteratively solves for internal ice temperature and ice thickness, using hybrid shallow-ice/shallow-shelf ice dynamics and a velocity-based grounding-line parameterisation^68^. These simplified physics enable computations spanning long timescales and compare reasonably well to higher-order models^69^. The PSU-ISM includes bedrock rebound in response to changes in ice loading, by modelling deformation as an elastic lithospheric plate above local isostatic relaxation. The model is able to represent marine ice-shelf instabilities^70^, although these mechanisms are only triggered in warmer-than-present climates and therefore do not significantly impact LGM-to-present simulations (e.g. ref. ^67^).

The model includes an internal calculation for bed-elevation change in response to ice loading change (via an elastic-lithosphere and viscously relaxing-asthenosphere module) and is forced by eustatic deglacial sea-level change, but is not coupled to a solid-Earth/sea-level model and thus does not simulate relative sea-level response to gravitational change. Gravitational effects would cause additional relative sea-level to fall in the Amundsen Sea region during the last deglaciation (e.g. ref. ^23^), potentially enhancing ice-shelf pinning.

As in ref. ^67^, deglacial atmospheric forcing is derived by scaling a present-day climatology (ALBMAP^71^) by a uniform cooling perturbation based on deep-sea δ^18^O^72^. Oceanic forcing is provided by a global coupled climate-ocean model simulation of the last 22 kyr^73^, eustatic sea-level variation is from ICE5G^74^. Further details of the model formulation can be found in refs. ^66,75^.

The nested domains receive 3-D ice thickness and ice-velocity boundary conditions at the domain edges every 500 years, provided by the continental simulations to be downscaled. The nesting domains encompass both the relevant exposure-age site locations as well as any fast-flow regions (for example, upstream glacial catchment regions) that need to be resolved in order to accurately capture deglacial behaviour. The model parameters varied in our ensemble are CSHELF (a basal sliding coefficient value for regions outside of the modern inversion product, with values from 10^5^, 10^6^, or 10^7^ [m yr^−1^ Pa^−2^, corresponding to “C” in Eq. (11) of ref. ^75^]), OCFACMULT (a sub-ice oceanic melt coefficient, with values 1, 3, and 5 [non-dimensional; corresponding to “K” in Eq. (17) of ref. ^66^]), and TLAPSEPRECIP (modifies the scaling of precipitation with temperature, with values 7, 10, and 35 that correspond to a Clausius-Clapyeron temperature-precipitation relationship). More details on nesting, initialisation, model setup, and model parameters can be found in ref. ^31^.

We selected three model ensemble members that score reasonably well against the terrestrial datasets for the Amundsen Sea region, balancing the float scores and exceedance scores calculated by ref. ^31^. We downscaled three different model ensemble members, with parameter combinations given by [CSHELF]_[OCFACMULT]_[TLAPSEPRECIP]: 5_2_7, 5_2_35, and 6_5_7. This repetition allowed us to ensure that the linkages we infer between terrestrial thinning patterns and regional deglacial characteristics, such as grounding-line retreat, are robust across different parameter combinations and thus different styles of modelled deglacial characteristics. The primary model output that we analyse and plot here is 5_2_7. The other model ensemble members revealed similar terrestrial-marine linkages in terms of grounding-line configurations that corresponded to rapid inland thinning. We use depth-averaged velocity here as a diagnostic to understand ice flow paths through the deglaciation.

From the ice sheet model simulation, we output and analyse the buttressing factor θ—described in ref. ^76^ and calculated by their Eq. (2)—to characterise the amount of back-stress provided by an ice shelf caused by pinning points or lateral forces acting further downstream. This buttressing factor θ is defined as the ratio of vertically averaged horizontal deviatoric stress normal to the grounding line. Here we plot and analyse θ (specifically, the norm of θ_u_ and θ_v_) as in ref. ^76^, although we have reversed end-member values for clarity so that a value of 1 indicates minimal buttressing and lower values correspond to greater resistive stresses exerted by the ice shelf.

## Supplementary information

Supplementary Information

Description of Additional Supplementary File

Supplementary Data 1

Supplementary Data 2

Supplementary Data 3

Supplementary Data 4

Supplementary Data 5

Supplementary Data 6

Supplementary Movie 1

Transparent Peer Review file

## Data Availability

The data that support the findings of this study are available as follows: Cosmogenic-nuclide data and exposure ages can be accessed from Nichols, K., Rood, D., Wilcken, K. & Johnson, J. Cosmogenic ^10^Be data and calculated surface exposure ages from nunataks adjacent to Pine Island Glacier in the Hudson Mountains, West Antarctica (Version 1.0) [Dataset]. NERC EDS UK Polar Data Centre. https://doi.org/10.5285/9ddd50c8-cd08-4afe-b245-f6891f1d9a3f (2023) and Nichols, K., Rood, D., Johnson, J., Wilcken, K. & Brown, K. Cosmogenic ^10^Be data and calculated surface exposure ages for 41 cobbles and boulders collected from nunataks adjacent to Larter and Lucchitta glaciers in the Hudson Mountains, West Antarctica (Version 1.0) [Dataset]. NERC EDS UK Polar Data Centre. https://doi.org/10.5285/de4a62bc-9c01-4aa3-9fd8-c64d706cfc13 (2025). Model output including model fields of ice-sheet thickness and velocity, bed topography, and calculations of ice-sheet volume and global sea-level equivalent can be accessed from Peters, S. & Halberstadt, A. R. Synchronous Holocene thinning of Pine Island Glacier and its tributaries influenced by ice-shelf unpinning [Dataset]. Zenodo. https://doi.org/10.5281/zenodo.19861145 (2026). Model output shown in Supplementary Fig. 3 can be accessed from Bett, D. Amundsen Sea sector ice-ocean model output focused on pinning point ungrounding in the Pine Island Glacier system [Dataset]. Zenodo. https://doi.org/10.5281/zenodo.21128994 (2026). Detailed maps of each nunatak site are freely available from ref. ^26^, Appendix A.

## Code availability

The ice-sheet model code version is from ref. ^77^. The model runs conducted here are downscaled simulations directly following ref. ^31^.

## References

[1] Hughes, T. J. The weak underbelly of the West Antarctic Ice-Sheet. *J. Glaciol.* **27**, 518–525 (1981).

[2] Vaughan, D. G. et al. New boundary conditions for the West Antarctic ice sheet: Subglacial topography beneath Pine Island Glacier. *Geophys. Res. Lett.* **33**, L09501 (2006).

[3] Konrad, H. et al. Uneven onset and pace of ice-dynamical imbalance in the Amundsen Sea Embayment, West Antarctica. *Geophys. Res. Lett.* **44**, 910–918 (2017).

[4] Mouginot, J., Rignot, E. & Scheuchl, B. Sustained increase in ice discharge from the Amundsen Sea Embayment, West Antarctica, from 1973 to 2013. *Geophys. Res. Lett.* **41**, 1576–1584 (2014).

[5] Wingham, D. J., Wallis, D. W. & Shepherd, A. Spatial and temporal evolution of Pine Island Glacier thinning, 1995-2006. *Geophys. Res. Lett.* **36**, L17501 (2009).

[6] Jacobs, S. S., Jenkins, A., Giulivi, C. F. & Dutrieux, P. Stronger ocean circulation and increased melting under Pine Island Glacier ice shelf. *Nat. Geosci.* **4**, 519–523 (2011).

[7] Pritchard, H. et al. Antarctic ice-sheet loss driven by basal melting of ice shelves. *Nature* **484**, 502–505 (2012). 22538614 10.1038/nature10968

[8] Rignot, E. et al. Four decades of Antarctic Ice Sheet mass balance from 1979–2017. *Proc. Natl. Acad. Sci. USA* **116**, 1095–1103 (2019). 30642972 10.1073/pnas.1812883116 PMC6347714

[9] Bett, D. T. et al. Coupled ice–ocean interactions during future retreat of West Antarctic ice streams in the Amundsen Sea sector. * Cryosphere* **18**, 2653–2675 (2024).

[10] van den Akker, T. et al. Present-day mass loss rates are a precursor for West Antarctic Ice Sheet collapse. * Cryosphere* **19**, 283–301 (2025).

[11] Naughten, K. A., Holland, P. R. & De Rydt, J. Unavoidable future increase in West Antarctic ice-shelf melting over the twenty-first century. *Nat. Clim. Change* **13**, 1222–1228 (2023). 10.1038/s41558-023-01818-x PMC13538064 42694133

[12] Hillenbrand, C.-D. et al. Grounding-line retreat of the West Antarctic Ice Sheet from inner Pine Island Bay. *Geology* **41**, 35–38 (2013).

[13] Smith, J. A. et al. New constraints on the timing of West Antarctic Ice Sheet retreat in the eastern Amundsen Sea since the Last Glacial Maximum. *Glob. Planet. Change* **122**, 224–237 (2014).

[14] Johnson, J. S. et al. Rapid thinning of Pine Island Glacier in the early Holocene. *Science* **343**, 999–1001 (2014). 24557837 10.1126/science.1247385

[15] Nichols, K. A. et al. Offshore-onshore record of Last Glacial Maximum-to-present grounding line retreat at Pine Island Glacier. *Geology* **51**, 1033–1037 (2023).

[16] Hillenbrand, C.-D. et al. West Antarctic Ice Sheet retreat driven by Holocene warm water incursions. *Nature* **547**, 43–48 (2017). 28682333 10.1038/nature22995 PMC5510715

[17] Rignot, E., Casassa, G., Gogineni, P., Rivera, A. & Thomas, R. Accelerated ice discharges from the Antarctic Peninsula following the collapse of the Larsen B ice shelf. *Geophys. Res. Lett.* **31**, L18401 (2004).

[18] Scambos, T. A., Bohlander, J. A., Shuman, C. A. & Skvarca, P. Glacier acceleration and thinning after ice shelf collapse in the Larsen B embayment, Antarctica. *Geophys. Res. Lett.* **31**, L18402 (2004).

[19] North, R. & Barrows, T. T. High-resolution elevation models of Larsen B glaciers extracted from 1960s imagery. *Nat. Sci. Rep.* **14**, 14536 (2024). 10.1038/s41598-024-65081-6 PMC11231284 38977717

[20] Jones, R. et al. Rapid Holocene thinning of an East Antarctic outlet glacier driven by marine ice sheet instability. *Nat. Commun.* **6**, 8910 (2015). 26608558 10.1038/ncomms9910 PMC4674764

[21] Stutz, J. et al. Mid-Holocene thinning of David Glacier, Antarctica: chronology and controls. * Cryosphere* **15**, 5447–5471 (2021).

[22] Pattyn, F., Huyghe, A., De Brabander, S. & De Smet, B. Role of transition zones in marine ice sheet dynamics. *J. Geophys. Res. Earth Surf.* **111**, F02004 (2006).

[23] Gomez, N., Weber, M. E., Clark, P. U., Mitrovica, J. X. & Han, H. K. Antarctic ice dynamics amplified by Northern Hemisphere sea-level forcing. *Nature* **587**, 600–604 (2020). 33239798 10.1038/s41586-020-2916-2

[24] Jeong, S., Howat, I. M. & Bassis, J. N. Accelerated ice shelf rifting and retreat at Pine Island Glacier, West Antarctica. *Geophys. Res. Lett.* **43**, 11720–11725 (2016).

[25] De Rydt, J., Reese, R., Paolo, F. S. & Gudmundsson, G. H. Drivers of Pine Island Glacier speed-up between 1996 and 2016. * Cryosphere* **15**, 113–132 (2021).

[26] Johnson, J. S. et al. Glacial geology of the Hudson Mountains, Amundsen Sea sector, West Antarctica. *Quat. Sci. Rev.* **350**, 109027 (2025).

[27] Balco, G. Glacier change and paleoclimate applications of cosmogenic-nuclide exposure dating. *Annu. Rev. Earth Planet. Sci.* **48**, 21–48 (2020).

[28] Buizert, C. et al. Antarctic surface temperature and elevation during the Last Glacial Maximum. *Science* **372**, 1097–1101 (2021). 34083489 10.1126/science.abd2897

[29] Suganuma, Y. et al. Regional sea-level highstand triggered Holocene ice sheet thinning across coastal Dronning Maud Land, East Antarctica. *Commun. Earth Environ.* **3**, 273 (2022).

[30] Sharma, A. Recent ice dynamics of outlet glaciers in Enderby Land, Kemp Land and Dronning Maud Land, East Antarctica, and their links to ocean-climate forcings between 1960s and 2024. Doctoral thesis, Durham University. Preprint at https://etheses.durham.ac.uk/id/eprint/16415/ (2025).

[31] Halberstadt, A. R. W. & Balco, G. Antarctic ice sheet model comparison with uncurated geological constraints shows that higher spatial resolution improves deglacial reconstructions. * Cryosphere* **20**, 931–961 (2026).

[32] Clark, P. U. et al. The last glacial maximum. *Science* **325**, 710–714 (2009). 19661421 10.1126/science.1172873

[33] Cohen, K., Harper, D., Gibbard, P. & Car, N. The ICS international chronostratigraphic chart this decade. *Episodes* **48**, 105–115 (2025).

[34] Jordan, T. A., Johnson, J. S., Riley, T. R., Conrad, E. & Carter, A. Subglacial geology and palaeo flow of Pine Island Glacier from combining glacial erratics with geophysics. *Commun. Earth Environ.* **6**, 826 (2025).

[35] Mas e Braga, M. et al. Nunataks as barriers to ice flow: implications for palaeo ice sheet reconstructions. * Cryosphere* **15**, 4929–4947 (2021).

[36] Johnson, J. S. et al. Comparing glacial-geological evidence and model simulations of ice sheet change since the last glacial period in the Amundsen Sea sector of Antarctica. *J. Geophys. Res. Earth Surface* **126**, e2020JF005827 (2021).

[37] Mawbey, E. M. et al. Ocean heat forced West Antarctic Ice Sheet retreat after the Last Glacial Maximum. *Nat. Commun.* **17**, 2079 (2026). 41651827 10.1038/s41467-026-68949-5 PMC12953795

[38] Suganuma, Y. et al. Antarctic ice-shelf collapse in Holocene driven by meltwater release feedbacks. *Nat. Geosci.* **18**, 1216–1223 (2025).

[39] Graham, A. G. et al. Flow and retreat of the Late Quaternary Pine Island-Thwaites palaeo-ice stream, West Antarctica. *J. Geophys. Res. Earth Surf.* **115**, F03025 (2010).

[40] Jakobsson, M. et al. Ice sheet retreat dynamics inferred from glacial morphology of the central Pine Island Bay Trough, West Antarctica. *Quat. Sci. Rev.* **38**, 1–10 (2012).

[41] Larter, R. D. et al. Reconstruction of changes in the Amundsen Sea and Bellingshausen Sea sector of the West Antarctic ice sheet since the Last Glacial Maximum. *Quat. Sci. Rev.* **100**, 55–86 (2014).

[42] Balco, G. et al. Reversible ice sheet thinning in the Amundsen Sea Embayment during the Late Holocene. *Cryosphere* **17**, 1787–1801 (2023).

[43] Dutrieux, P. et al. Strong sensitivity of Pine Island Ice-Shelf melting to climatic variability. *Science* **343**, 174–178 (2014). 24385606 10.1126/science.1244341

[44] Muto, A. et al. Subglacial bathymetry and sediment distribution beneath Pine Island Glacier ice shelf modeled using aerogravity and in situ geophysical data: New results. *Earth Planet. Sci. Lett.* **433**, 63–75 (2016).

[45] Millan, R., Rignot, E., Bernier, V., Morlighem, M. & Dutrieux, P. Bathymetry of the Amundsen Sea Embayment sector of West Antarctica from Operation IceBridge gravity and other data. *Geophys. Res. Lett.* **44**, 1360–1368 (2017).

[46] Arndt, J. E. et al. Bathymetric controls on calving processes at Pine Island Glacier. * Cryosphere* **12**, 2039–2050 (2018).

[47] Hogan, K. A. et al. Revealing the former bed of Thwaites Glacier using sea-floor bathymetry: implications for warm-water routing and bed controls on ice flow and buttressing. *Cryosphere* **14**, 2883–2908 (2020).

[48] Matsuoka, K. et al. Antarctic ice rises and rumples: Their properties and significance for ice-sheet dynamics and evolution. *Earth Sci. Rev.* **150**, 724–745 (2015).

[49] Holland, P. R. et al. Anthropogenic and internal drivers of wind changes over the Amundsen Sea, West Antarctica, during the 20th and 21st centuries. *Cryosphere* **16**, 5085–5105 (2022).

[50] Braddock, S. et al. Relative sea-level data preclude major late Holocene ice-mass change in Pine Island Bay. *Nat. Geosci.* **15**, 568–572 (2022).

[51] Book, C. et al. Stabilizing effect of bedrock uplift on retreat of Thwaites Glacier, Antarctica, at centennial timescales. *Earth Planet. Sci. Lett.* **597**, 117798 (2022).

[52] Johnson, J. S., Bentley, M. J. & Gohl, K. First exposure ages from the Amundsen Sea Embayment, West Antarctica: the Late Quaternary context for recent thinning of Pine Island, Smith and Pope Glaciers. *Geology* **36**, 223–226 (2008).

[53] Kohl, C. P. & Nishiizumi, K. Chemical isolation of quartz for measurement of in-situ -produced cosmogenic nuclides. *Geochim. Cosmochim. Acta* **59**, 3583–3587 (1992).

[54] Herber, L. J. Separation of feldspar from quartz by flotation. *Am. Miner.* **54**, 1212–1215 (1969).

[55] Corbett, L. B. et al. An approach for optimizing in situ cosmogenic ^10^Be sample preparation. *Quat. Geochronol.* **33**, 24–34 (2016).

[56] Jull, A. J. T., Scott, E. M. & Bierman, P. The CRONUS-Earth inter-comparison for cosmogenic isotope analysis. *Quat. Geochronol.* **26**, 3–10 (2015).

[57] Wilcken, K. et al. Accelerator Mass Spectrometry on SIRIUS: New 6 MV spectrometer at ANSTO. *Nucl. Instrum. Methods Phys. Res. Sect. B Beam Interact. Mater. At.* **406**, 278–282 (2017).

[58] Wilcken, K. M. et al. Technical note: Accelerator mass spectrometry of ^10^Be and ^26^Al at low nuclide concentrations. *Geochronology* **4**, 339–352 (2022).

[59] Nishiizumi, K. et al. Absolute calibration of ^10^Be AMS standards. *Nucl. Instrum. Methods Phys. Res. Sect. B.* **258**, 403–413 (2007).

[60] Marschalek, J. W. et al. Geological insights from the newly discovered granite of Sif Island between Thwaites and Pine Island glaciers. *Antarct. Sci.* **36**, 51–74 (2024).

[61] Balco, G., Stone, J. O., Lifton, N. A. & Dunai, T. J. A complete and easily accessible means of calculating surface exposure ages or erosion rates from ^10^Be and ^26^Al measurements. *Quat. Geochronol.* **3**, 174–195 (2008).

[62] Borchers, B. Geological calibration of spallation production rates in the CRONUS-Earth project. *Quat. Geochronol.* **31**, 188–198 (2016).

[63] Lifton, N. A., Sato, T. & Dunai, T. J. Scaling in situ cosmogenic nuclide production rates using analytical approximations to atmospheric cosmic-ray fluxes. *Earth Planet. Sci. Lett.* **386**, 149–160 (2014).

[64] Balco, G. Production rate calculations for cosmic-ray-muon-produced ^10^Be and ^26^Al benchmarked against geological calibration data. *Quat. Geochronol.* **39**, 150–173 (2017).

[65] Stone, J. O. Air pressure and cosmogenic isotope production. *J. Geophys. Res. Solid Earth* **105**, 23753–23759 (2000).

[66] Pollard, D. & DeConto, R. M. A simple inverse method for the distribution of basal sliding coefficients under ice sheets, applied to Antarctica. * Cryosphere* **6**, 953–971 (2012b).

[67] Pollard, D., Chang, W., Haran, M., Applegate, P. & DeConto, R. Large ensemble modeling of last deglacial retreat of the West Antarctic Ice Sheet: comparison of simple and advanced statistical techniques. *Geosci. Model Devel.* **9**, 1697–1723 (2016).

[68] Schoof, C. Ice sheet grounding line dynamics: steady states, stability, and hysteresis. *J. Geophys. Res. Earth* **112**, F03S28 (2007).

[69] Cornford, S. L. et al. Results of the third Marine Ice Sheet Model Intercomparison Project (MISMIP+). * Cryosphere* **14**, 2283–2301 (2020).

[70] Pollard, D., DeConto, R. M. & Alley, R. B. Potential Antarctic Ice Sheet retreat driven by hydrofracturing and ice cliff failure. *Earth Planet. Sci. Lett.* **412**, 112–112 (2015).

[71] Le Brocq, A. M., Payne, A. J. & Vieli, A. An improved Antarctic dataset for high resolution numerical ice sheet models (ALBMAP v1). *Earth Syst. Sci. Data* **2**, 247–260 (2010).

[72] Lisiecki, L. E. & Raymo, M. E. A Pliocene-Pleistocene stack of 57 globally distributed benthic δ^18^O records. *Paleoceanogr. Paleoclimatol.* **20**, PA1003 (2005).

[73] Liu, Z. et al. Transient simulation of last deglaciation with a new mechanism for Bolling-Allerod warming. *Science* **325**, 310–314 (2009). 19608916 10.1126/science.1171041

[74] Peltier, W. R. Global glacial isostacy and the surface of the Ice-Age Earth. *Ann. Rev. Earth Planet. Sci.* **32**, 111–149 (2004).

[75] Pollard, D. & DeConto, R. M. Description of a hybrid ice sheet-shelf model, and application to Antarctica. *Geosci. Model Devel.* **5**, 1273–1295 (2012a).

[76] Pollard, D. & DeConto, R. M. Improvements in one-dimensional grounding-line parameterizations in an ice-sheet model with lateral variations (PSUICE3D v2.1). *Geosci. Model Dev.* **13**, 6481–6500 (2020).

[77] DeConto, R. M. et al. The Paris Climate Agreement and future sea level rise from Antarctica. *Nature* **593**, 83–89 (2021). 33953408 10.1038/s41586-021-03427-0

[78] Dorschel, B. et al. The International Bathymetric Chart of the Southern Ocean Version 2 (IBCSO v2) [Dataset]. PANGAEA, 10.1594/PANGAEA.937574 (2022). PMC9174482 35672417

[79] Rignot, E., Mouginot, J. & Scheuchl, B. MEaSUREs InSAR-Based Antarctica Ice Velocity Map, Version 2. Boulder, Colorado USA, NASA National Snow and Ice Data Center Distributed Active Archive Center. 10.5067/D7GK8F5J8M8R (2017).

[80] Bindschadler, R. et al. The Landsat Image Mosaic of Antarctica. *Remote Sens. Environ.* **112**, 4214–4226 (2008).

[81] Moholdt, G., Pritchard, H., & Maton, J. RINGS/Bedmap3 grounding line of the Antarctic Ice Sheet [Dataset]. Norwegian Polar Institute, 10.21334/NPOLAR.2025.D1062D2A (2025).

[82] Gerrish, L., Ireland, L., Fretwell, P. & Cooper, P. Medium resolution vector polylines of the Antarctic coastline (Version 7.10) [Dataset]. NERC EDS UK Polar Data Centre. 10.5285/bc81931c-4e8e-439a-b3c9-d3d1fdb109df (2024).

