# Supplementary material for: Synchronous Holocene thinning of Pine Island Glacier and its tributaries influenced by ice-shelf unpinning
Source: Nat Commun. 2026 Aug 11;17:9639. doi: 10.1038/s41467-026-76244-6 (PMC13554069; doi:10.1038/s41467-026-76244-6)
Supplement: Supplementary file 1 [file 41467_2026_76244_MOESM10_ESM.pdf]

nature portfolio

Peer Review File

Synchronous Holocene thinning of Pine Island Glacier and its tributaries influenced by ice-shelf unpinning

Corresponding Author: Dr Joanne Johnson

This file contains all reviewer reports in order by version, followed by all author rebuttals in order by version.

Version 0:

Reviewer comments:

Reviewer #1

(Remarks to the Author)
Reviewer Report

1. Summary and General Comments

This manuscript presents a high-quality investigation into the Holocene thinning of Pine Island Glacier (PIG) and its tributaries by integrating Be-10 exposure dating with high-resolution numerical modeling. The extensive dataset—comprising 82 exposure ages, including 41 new data points—provides a significant contribution to our understanding of the history of the Amundsen Sea sector. The authors' focus on the synchronous nature of thinning during the mid-Holocene and its link to ice-shelf unpinning is a compelling and timely topic.

While the results are impressive, I have several recommendations to improve the clarity of the narrative and the technical transparency of the model. Most importantly, I suggest that the authors more explicitly define the fundamental scientific questions at the outset of the paper. Although the results are excellent, the manuscript would benefit from a clearer roadmap in the introduction: what specific gaps in current understanding existed regarding tributary synchronicity, and how was this study's integrated approach specifically designed to address them? Providing this context early on will help the reader better appreciate the significance and impact of the findings.

2. Specific Comments

Consideration of Relative Sea-Level (RSL) Change in Modeling:
The authors emphasize that ice-shelf unpinning was the primary trigger for the synchronous thinning at 7.1 ka. However, the timing of such an unpinning event is physically coupled not only to ice thickness but also to relative sea level (RSL) at the pinning points. It is currently unclear whether the numerical model incorporates time-evolving RSL (accounting for both eustatic changes and local GIA-induced crustal uplift). Given that RSL variations directly influence the buoyancy and grounding stability of the ice shelf over bedrock highs, the authors should clarify their assumptions regarding RSL and discuss how variations in sea level might have influenced the timing of the observed thinning.

Clarification of "Local Ice Cap" Signals:
The discrepancy observed at high-elevation sites such as Mount Moses, where exposure ages are approximately 5,000 years older than the model predictions, is currently attributed to model limitations. However, these older ages may instead reflect the retreat of a local ice cap independent of the main PIG trunk thinning. Please provide geomorphological evidence to distinguish between the regional ice-sheet signal and local ice-cap retreat, and discuss how this affects your interpretation of the "main trunk" thickness.

Discussion of Regional Context:
The discussion of ocean–ice interactions and ice-sheet retreat could be further strengthened by placing the results in a broader regional context. For example, it may be worth mentioning how your findings regarding the timing of thinning relate to the environmental transitions and oceanic influences discussed in Suganuma et al. (2025). Briefly highlighting how these

different lines of evidence complement each other could enhance the regional relevance of the study.

Originality of Figure 1:
I noticed that Figure 1 appears to be identical or very similar to a figure used in the author's previous publication. To ensure the originality of this manuscript and to better reflect the specific focus of this new study, I recommend revising or updating Figure 1. For instance, incorporating new data points or emphasizing the specific tributary domains discussed in this work would be more appropriate.

Missing Model Parameters in Fig. 1 and Text:
While Fig. 1 and the accompanying text discuss ice flow and velocity, the specific parameters for advection speed (or the velocity-field configuration) are not described in sufficient detail. To allow for a proper evaluation of the model's physical consistency and its ability to propagate thinning signals upstream, these parameters should be explicitly stated in the Methods section or provided in a table.

Requirement for Detailed Maps of Sampling Sites:
To better evaluate the geological and geomorphological context of the results, please provide detailed, larger-scale maps for each sampling location. These maps should clarify the relationship between sample positions, local topography, and the main ice-flow paths, which would significantly help in assessing potential local influences.

Improvement of Figure 5:
Figure 5 is currently difficult to interpret due to limited visibility. The overlapping streamlines and the contrast between background bed elevation and velocity fields make it challenging to discern the primary trends. I recommend revising the color scheme and layout to improve clarity, ensuring that the convergence of flowlines and grounding-line positions is easily identifiable.

Reviewer #2

(Remarks to the Author)
This paper combines existing and new cosmogenic nuclide exposure data from the Hudson Mountains, in the catchment of Pine Island Glacier. These data show a largely synchronous timing of retreat during the Holocene. These data are supplemented by ice sheet modelling experiments of the deglaciation for the same catchment, which highlight the role of downstream pinning points and buttressing on the rate of glacier thinning. The title is careful to highlight that the timing of glacier is 'influenced' by ice shelf unpinning, rather than being the central factor. I think this is wise as although unpinning is likely an important process it is hard to disentangle if this was the cause of the synchronous thinning versus other regional forcing (e.g. climate, ocean or sea level forcing). This is similarly the case for the modelling experiments. Overall, this is an impressive study that nicely combines field data with ice sheet modelling. The results are novel and it is a valuable contribution that is well written and presented, I only have a few suggested changes.

1) The new data are not clearly differentiated from data that has been previously published. These should be clearly labelled on Figure 1 & 2, e.g. by including unfilled/filled markers on Fig 2. It took be a while to work out which of the data are new. The introduction could have stronger motivation as to why these additional data were needed.

2) It's difficult to determine what role ice shelf buttressing had on the timing of thinning. Because there is synchronous thinning it is suggested that it must be due to regional-scale processes and shared boundary conditions, rather than local effects (e.g. topography). The modelling is used to show that the pinning points cause a slow-down and then acceleration in thinning, but this isn't clearly seen in the data. So, another regional-scale process could also cause the synchronous thinning. As multiple processes are changing in the model it is difficult to determine which is the more important process. One option would be to run the simulations with the pinning points removed, e.g. by lowering the topography that the ice shelf pins on, this would more clearly show the effect of pinning/unpinning on the rate of thinning. This is a suggestion, I think the paper stands on its own without these additional simulations, but it could be made stronger with their inclusion.

Specific points:

L203: I got very confused by this sentence and the reasoning behind it. Could you not just state that the model generates a similar pattern/timing across the glacier system and then clarify that just one glacier is shown in Fig. 3a.

Figure 4: To compare the model and data could you also show this with the exposure age for each as simulated by the model? Or as model-data residuals? This figure is confusing as you need to line up the specific glacier exposure data with the equivalent modelled glacier.

L106: When trying to work out which are the new sites I could only see 14 unique sites – is this because one is very close to another so doesn't show up on the map? Similarly in the attached excel file.

L125: What is the claim that this is an order of magnitude improvement on existing studies based on?

Version 1:

Reviewer comments:

Reviewer #1

(Remarks to the Author)

The authors have addressed all comments satisfactorily. The revisions improve the clarity of the Introduction, the transparency of the model description (RSL treatment, velocity field), and the geomorphological justification for interpreting the exposure ages as a regional ice-sheet signal rather than a local ice-cap signal.
I recommend acceptance of the manuscript.

Reviewer #2

(Remarks to the Author)
The authors have done a good job in responding to mine and the other reviewer's previous comments, this is a very useful study and I have no further comments on the revised manuscript.

Open Access This Peer Review File is licensed under a Creative Commons Attribution 4.0 International License, which permits use, sharing, adaptation, distribution and reproduction in any medium or format, as long as you give appropriate credit to the original author(s) and the source, provide a link to the Creative Commons license, and indicate if changes were made.
In cases where reviewers are anonymous, credit should be given to 'Anonymous Referee' and the source.
The images or other third party material in this Peer Review File are included in the article's Creative Commons license, unless indicated otherwise in a credit line to the material. If material is not included in the article's Creative Commons license and your intended use is not permitted by statutory regulation or exceeds the permitted use, you will need to obtain permission directly from the copyright holder.
To view a copy of this license, visit https://creativecommons.org/licenses/by/4.0/

Reviewer #1:

1. Summary and General Comments

This manuscript presents a high-quality investigation into the Holocene thinning of Pine Island Glacier (PIG) and its tributaries by integrating Be-10 exposure dating with high-resolution numerical modeling. The extensive dataset—comprising 82 exposure ages, including 41 new data points—provides a significant contribution to our understanding of the history of the Amundsen Sea sector. The authors' focus on the synchronous nature of thinning during the mid-Holocene and its link to ice-shelf unpinning is a compelling and timely topic.

While the results are impressive, I have several recommendations to improve the clarity of the narrative and the technical transparency of the model. Most importantly, I suggest that the authors more explicitly define the fundamental scientific questions at the outset of the paper. Although the results are excellent, the manuscript would benefit from a clearer roadmap in the introduction: what specific gaps in current understanding existed regarding tributary synchronicity, and how was this study's integrated approach specifically designed to address them? Providing this context early on will help the reader better appreciate the significance and impact of the findings.

Thank you for raising this point. We agree that a clearer roadmap would be useful in the Introduction, particularly in the opening paragraph of the paper so that readers are clear about the scope of our study from the outset. We have thus amended the Introduction at lines 43-48, and 123-133, adding sentences both to outline the aims and methods of our study and to provide some context on temporal variability of ice-sheet thinning. We have decided not to describe "gaps in current understanding" regarding synchronicity, as suggest, because our data-model comparison study was designed to understand the broader relationship between Holocene grounding-line dynamics and rapid ice-sheet thinning in the Pine Island Glacier system, regardless of whether or not thinning was found to be synchronous across the system. The synchronicity was something we discussed only when we obtained the new exposure-age data. We have also added text to the Introduction at lines 103-106 to clarify for readers what the new data provide – that is, deglacial thinning histories for Larter and Lucchitta glaciers (to add to that already published from Pine Island Glacier), and more data from higher elevation nunataks.

2. Specific Comments

Consideration of Relative Sea-Level (RSL) Change in Modeling:
The authors emphasize that ice-shelf unpinning was the primary trigger for the synchronous thinning at 7.1 ka. However, the timing of such an unpinning event is physically coupled not only to ice thickness but also to relative sea level (RSL) at the pinning points. It is currently unclear whether the numerical model incorporates time-evolving RSL (accounting for both eustatic changes and local GIA-induced crustal uplift). Given that RSL variations directly influence the buoyancy and grounding stability of the ice shelf over bedrock highs, the authors should clarify their assumptions regarding RSL and discuss how variations in sea level might have influenced the timing of the observed thinning.

We have added text into the Methods section at lines 601-606 to clarify that the model does represent eustatic sea-level change and GIA-induced crustal uplift.

Clarification of “Local Ice Cap” Signals:
The discrepancy observed at high-elevation sites such as Mount Moses, where exposure ages are approximately 5,000 years older than the model predictions, is currently attributed to model limitations. However, these older ages may instead reflect the retreat of a local ice cap independent of the main PIG trunk thinning. Please provide geomorphological evidence to distinguish between the regional ice-sheet signal and local ice-cap retreat, and discuss how this affects your interpretation of the “main trunk” thickness.

It is correct that there is a mismatch between the model predictions and exposure ages at Mount Moses, but as mentioned in lines 297-300, the exposure ages are in fact 5,000 years *younger*, not older, than the model predictions (see Fig. 4b). In our response below, we have therefore assumed that you meant to write “younger”.

Firstly, we think it is important to clarify what local versus regional ice cover might have looked like in the past. We interpret “local” ice as being either glaciers on the summit of an individual nunatak or a broader ice cap covering groups of nunataks. The effect of retreat on the exposure-age profiles in each of these cases would be different.

We made the following relevant geomorphic observations at the Hudson Mountains sites (see also Johnson et al., 2025, cited here as ref. 26):

(i) there are no moraines that would be indicative of locally fed glaciers on any nunatak
(ii) at every site, the exposure ages get younger downhill (not uphill as would be expected if they reflected retreat of locally fed glaciers)
(iii) although striations were very occasionally observed on bedrock surfaces near the ice margin, except at Webber Nunatak (see below) none of those striations are oriented radially outward from the nunatak summits and none were observed on the summits or at higher elevations (if present, these would indicate an ice cap)
(iv) erratics are of exotic lithology (granitoids and gabbro), indicating a source not local to the nunataks (which are, in contrast, composed of basaltic lava, hyaloclastite and tuff) – this implies that the exposure ages here reflect the regional ice-sheet signal, not retreat of local ice

Thus, the observed geomorphic evidence is not consistent with the presence of locally fed glaciers or a broader ice cap in the Hudson Mountains, except perhaps at Webber Nunatak which is the only nunatak that does not have erratics on its summit (see Johnson et al., 2025 for detailed discussion).

Regarding the model’s ability to distinguish between local versus regional ice-sheet changes, development of a local ice cap whose thickness was controlled by ice flow from local glaciers rather than trunk glacier thinning is a process that would be simulated by the model if it were glaciologically feasible and favoured by the boundary conditions. If a very small local ice cap that was too small to be resolved by the model existed, this would be identifiable by the exposure ages younging uphill; we do not see this pattern at any sites in the Hudson Mountains. Thus, we are confident that the young exposure ages at high elevations on Mount Moses reflect regional thinning (of the main glacier trunks) rather than later deglaciation of a local ice cap or locally fed glaciers, and that model limitations remain a more likely reason for the observed data-model mismatch.

To provide clarity on these points, we have added a summary of the geomorphic evidence described above to the text at lines 189-195 and have pointed out that exposure ages at all the sites get younger downhill by adding a few words to line 164. We have also added a sentence at lines 297-300 where we discuss implications for our interpretation of the model simulations.

Discussion of Regional Context:
The discussion of ocean–ice interactions and ice-sheet retreat could be further strengthened by placing the results in a broader regional context. For example, it may be worth mentioning how your findings regarding the timing of thinning relate to the environmental transitions and oceanic influences discussed in Suganuma et al. (2025). Briefly highlighting how these different lines of evidence complement each other could enhance the regional relevance of the study.

The intention of our section on “regional context” – starting in line 320 – is to focus on the Amundsen Sea region of West Antarctica because this provides the immediate regional context for our PIG system study. Suganuma et al. (2025) https://www.nature.com/articles/s41561-025-01829-7 focused on a region of East Antarctica (Lützow-Holm Bay) which is more than 3,500 kilometres away from the Amundsen Sea region. We therefore think that expanding this section to discuss the East Antarctic Ice Sheet would make it too broad. It would also require us to include descriptions of regions elsewhere in Antarctica. Nevertheless, we accept that the inflow of warm Circumpolar Deep Water and ice-shelf collapse as triggers for inland thinning are important processes relevant to both regions. Thus, to address the above comment, we have added sentences at lines 336-343 that discuss these processes and cite Suganuma et al. (2025) whilst maintaining the focus on the Amundsen Sea sector. In addition, we have cited Mawbey et al. (2026) https://www.nature.com/articles/s41467-026-68949-5, which was published since we submitted our original manuscript and is highly relevant to our study both in topic and region – it describes the role of the ocean in driving Holocene ice-sheet retreat and thinning in the Amundsen Sea sector.

Originality of Figure 1:
I noticed that Figure 1 appears to be identical or very similar to a figure used in the author's previous publication. To ensure the originality of this manuscript and to better reflect the specific focus of this new study, I recommend revising or updating Figure 1. For instance, incorporating new data points or emphasizing the specific tributary domains discussed in this work would be more appropriate.

We deliberately chose the same colour scheme and similar layout for these maps as in previous publications to facilitate easy cross-reference, and also to maintain colour-blind friendliness (note however that the grounding line and coastline are updated here to reflect recent changes in the region) - we wish to keep this consistency here. However, we accept that the new data sites should be easy to differentiate and have therefore revised Figure 1, outlining in bold those sites which have new exposure age data (this also addresses Reviewer 2’s comment below). In addition, we added the tributary domains as suggested. We have also revised Figure 2 to highlight the exposure ages obtained from the new samples.

Missing Model Parameters in Fig. 1 and Text:
While Fig. 1 and the accompanying text discuss ice flow and velocity, the specific parameters for advection speed (or the velocity-field configuration) are not described in sufficient detail. To allow for a proper evaluation of the model’s physical consistency and its ability to propagate thinning signals upstream, these parameters should be explicitly stated in the Methods section or provided in a table.

We have added text to the Methods section at lines 589-591 and 633-634 to provide further detail on this. The model issues a hybrid SSA-SIA (Shallow Shelf Approximation-Shallow Ice Approximation) stress balance approach, documented in Pollard & DeConto (2012a; ref. 75). None of the parameters that are varied by Halberstadt & Balco (2026) [ref. 31] – who

generated the model ensemble where the simulations we use here were originally produced – impact the model's ability to laterally transmit stresses.

Requirement for Detailed Maps of Sampling Sites:
To better evaluate the geological and geomorphological context of the results, please provide detailed, larger-scale maps for each sampling location. These maps should clarify the relationship between sample positions, local topography, and the main ice-flow paths, which would significantly help in assessing potential local influences.

We did not include large-scale maps for each sampling location in this manuscript because detailed georeferenced maps of each nunatak are already published and openly accessible in the supplement to the following paper, which also describes the geomorphology of all the sites in detail: https://doi.org/10.1016/j.quascirev.2024.109027 (see Figures S10-S25). In those maps, the local topography (ice and rock) is indicated by contours overlain on high-resolution satellite imagery and the maps also show locations of all samples whose surface exposure age has been determined. We therefore think it is unnecessary to include any new maps here. To signpost readers to the published maps, we have added a sentence to the Data Availability section (line 666).

Improvement of Figure 5:
Figure 5 is currently difficult to interpret due to limited visibility. The overlapping streamlines and the contrast between background bed elevation and velocity fields make it challenging to discern the primary trends. I recommend revising the color scheme and layout to improve clarity, ensuring that the convergence of flowlines and grounding-line positions is easily identifiable.

We agree this figure is not clear in the original manuscript. As suggested, we have redrafted it to improve overall clarity and the visibility of the flowlines. We have changed the layout (scale bars now shown below the main panels, not vertically along the sides) and selected a colour-blind friendly palette for the sample sites, flowlines and grounding lines. For continuity, we also matched the colour ramps for the bed elevation and velocity fields with those in Figure 6 (this required minor modifications to Figure 6). Since the flowlines here in Figure 5 converge in a small area, some of their individual paths appear in the figure to overlap. However, the general trends are discernible, which is what this figure is intended to convey.

Reviewer #2:

This paper combines existing and new cosmogenic nuclide exposure data from the Hudson Mountains, in the catchment of Pine Island Glacier. These data show a largely synchronous timing of retreat during the Holocene. These data are supplemented by ice sheet modelling experiments of the deglaciation for the same catchment, which highlight the role of downstream pinning points and buttressing on the rate of glacier thinning. The title is careful to highlight that the timing of glacier is 'influenced' by ice shelf unpinning, rather than being the central factor. I think this is wise as although unpinning is likely an important process it is hard to disentangle if this was the cause of the synchronous thinning versus other regional forcing (e.g. climate, ocean or sea level forcing). This is similarly the case for the modelling experiments. Overall, this is an impressive study that nicely combines field data with ice sheet modelling. The results are novel and it is a valuable contribution that is well written and presented, I only have a few suggested changes.

We are grateful for these supportive comments, thank you. We address the suggested changes below.

1) The new data are not clearly differentiated from data that has been previously published. These should be clearly labelled on Figure 1 & 2, e.g. by including unfilled/filled markers on Fig 2. It took be a while to work out which of the data are new. The introduction could have stronger motivation as to why these additional data were needed.

We apologise for the confusion here. To differentiate the new data, in Figure 1b we have used bold font for the placenames and thickened the outlines for the symbols representing the new data sites, and amended the figure caption accordingly. Likewise, we have revised Figure 2 and its caption so that the new data are now shown as dark grey symbols and the sites with new data are indicated with bold names. We have also provided justification for the new data in the Introduction of the revised manuscript (see our response to Reviewer #1's initial comment).

2) It's difficult to determine what role ice shelf buttressing had on the timing of thinning. Because there is synchronous thinning it is suggested that it must be due to regional-scale processes and shared boundary conditions, rather than local effects (e.g. topography). The modelling is used to show that the pinning points cause a slow-down and then acceleration in thinning, but this isn't clearly seen in the data. So, another regional-scale process could also cause the synchronous thinning. As multiple processes are changing in the model it is difficult to determine which is the more important process. One option would be to run the simulations with the pinning points removed, e.g. by lowering the topography that the ice shelf pins on, this would more clearly show the effect of pinning/unpinning on the rate of thinning. This is a suggestion, I think the paper stands on its own without these additional simulations, but it could be made stronger with their inclusion.

We agree that other processes could drive synchronous thinning. Our goal here is to leverage existing modelling work to contextualise the processes that are probably important. The aim of our model analysis is therefore to show that the buttressing effect of pinning points is glaciologically plausible and even probable, given the mapped seafloor bedrock highs. Overall, this work is intended as a model-supported data interpretation, rather than a modelling sensitivity study, and a broader model ensemble falls outside of the scope of such data-based work.

Specific points:

L203: I got very confused by this sentence and the reasoning behind it. Could you not just state that the model generates a similar pattern/timing across the glacier system and then clarify that just one glacier is shown in Fig. 3a.

We think the sentence in line 198 (of the original manuscript) is what is referred to here, not line 203. We agree that it is not well-phrased and have therefore revised it accordingly (see lines 236-237, and a small clarification added in line 231).

Figure 4: To compare the model and data could you also show this with the exposure age for each as simulated by the model? Or as model-data residuals? This figure is confusing as you need to line up the specific glacier exposure data with the equivalent modelled glacier.

We acknowledge that there is a large amount of data visualized in Figure 4. We have spent much time considering how best to present the details of our large exposure-age dataset alongside the model outputs, and decided that this is the simplest way of achieving our goal for the figure – which is to broadly visualise the modelled versus measured thinning behaviour across the three glacier catchments whilst also showing thinning profiles at individual nunatak sites.

To compile the figure, we extracted the deglacial thinning curve for each model grid cell corresponding to each nunatak sampling site. We think this is the equivalent of the “exposure age for each as simulated by the model” in the comment above. As discussed in Halberstadt & Balco (2026; ref. 31), aligning thinning histories derived from exposure ages with modelled thinning histories is subject to many assumptions and complexities. Thus, we decided not to calculate a model-data mismatch, or ‘residual’, because that is complicated and may not be meaningful in this context.

L106: When trying to work out which are the new sites I could only see 14 unique sites – is this because one is very close to another so doesn’t show up on the map? Similarly in the attached excel file.

We apologise for this error. There are indeed 14 unique nunatak sites for which we have exposure ages, as the reviewer suggests (our error was triggered by the fact that we visited a fifteenth nunatak, World’s End Bluff, but did not date any samples from there). We have corrected the manuscript accordingly in lines 118, 157-8 and provided further clarification in the Methods at lines 493-498. We have also identified the new sites on Figures 1 and 2. The original submitted excel file (Supplementary Table 1) is correct.

L125: What is the claim that this is an order of magnitude improvement on existing studies based on?

This sentence was intended to explain what improvements are made by using nested ice-sheet models, but we agree it did not do a good job in this regard. In the revised manuscript, we have therefore rephrased the sentence (in lines 145-147) to better clarify the improvements made by using nested simulations rather than referring vaguely to “existing studies”.
