# Supplementary material for: Synchronous Holocene thinning of Pine Island Glacier and its tributaries influenced by ice-shelf unpinning
Source: Nat Commun. 2026 Aug 11;17:9639. doi: 10.1038/s41467-026-76244-6 (PMC13554069; doi:10.1038/s41467-026-76244-6)
Supplement: Supplementary file 2 [file 41467_2026_76244_MOESM1_ESM.pdf]

Supplementary Information

This document contains supplementary information for the following paper:

Synchronous Holocene thinning of Pine Island Glacier and its tributaries influenced by ice-shelf unpinning

by Joanne S. Johnson, Sara C. Peters, Keir A. Nichols, Anna Ruth Halberstadt, Katie Brown, Dylan H. Rood, Klaus Wilcken, David Bett, Jonathan R. Adams, Greg Balco, John Woodward

We provide the following:

Supplementary text

This document, including:

Determination of relative ice-surface elevations

Supplementary Figure 1. Kernel density plot showing clustering of Hudson Mountains exposure ages.

Supplementary Figure 2. Satellite imagery and bathymetry of inner Pine Island Bay.

Supplementary Figure 3. Results of coupled ice-ocean model simulation of the PIG system.

Supplementary Table 1. Observed and modelled average ice-sheet thinning rates (m yr^{-1}) in the Hudson Mountains region.

Supplementary Movie 1. Modelled thinning at nunataks in the Hudson Mountains through the last 15 kyrs. The left-hand panel shows rate of grounded-ice thickness change (dh/dt). Circles represent nunatak locations and are coloured according to the glacier whose thinning history they reflect (see text). Solid and dashed lines in the right-hand panel are the modelled ice-sheet thinning profiles at each nunatak, with the lines coloured according to the related glacier, as in the left-hand panel. [separate MP4 file]

Supplementary Data 1. All ^{10}Be exposure ages from Hudson Mountains (i.e. published ages, and new ages presented for the first time in this paper). [separate MS Excel spreadsheet]

Supplementary Data 2. Sample information from new analyses required for exposure age calculation (latitude, longitude, elevation, topographic shielding correction factor, and sample thickness). [separate MS Excel spreadsheet]

Supplementary Data 3. Analytical data and exposure ages (including $^{10}Be/^{9}Be$ ratios and ^{10}Be concentration) for the new analyses. [separate MS Excel spreadsheet]

Supplementary Data 4. Procedural blanks for the new analyses. [separate MS Excel spreadsheet]

Supplementary Data 5. Information formatted for online exposure age calculator input, for new analyses. [separate MS Excel spreadsheet]

Supplementary Data 6. Relative ice-surface elevations and locations used to define them. [separate MS Excel spreadsheet]

Determination of relative ice-surface elevations

In a previous study[1], relative ice-surface elevations for nunataks sampled for cosmogenic nuclide analyses adjacent to Pine Island Glacier —Evans Knoll, Winkie Nunatak, Shepherd Dome, Inman Nunatak, and Meyers Nunatak— were defined in two different ways to provide as much information as possible to potential users, particularly numerical ice-sheet modellers:

1. Ice elevation defined relative to the elevation of a point on the approximate centreline of Pine Island Glacier perpendicular to each sample site, with that elevation sourced from the Reference Model of Antarctica (REMA)[2].
2. 'Local' ice elevation defined using a combination of REMA, satellite imagery, and field observations to identify a relative ice elevation more proximal to each study site.

A later study[3] used the same approach to define relative ice elevations for sites adjacent to Larter Glacier and a tributary of Lucchitta Glacier using the location on an adjacent glacier centreline but did not repeat the method for identifying the more nuanced, local relative ice elevation. Here, we use the second approach to define local ice elevations for nunataks adjacent to Larter Glacier and a tributary of Lucchitta Glacier. The rationale used to obtain each ice elevation is described below.

At Webber Nunatak, the local ice elevation is defined where there is a break in the ice-surface slope at the bottom of the ridge from which the erratic cobbles were collected. At Mount Manthe, we use the ice surface at the base of a cliff facing Larter Glacier (avoiding a meltwater-filled windscoop). At Maish Nunatak, we use a break in slope on the ice surface that extends away from the westernmost exposed ridge (facing Larter Glacier). There is a prominent windscoop on the Larter Glacier side of the western end of Mount Moses. Therefore, to define a local ice elevation at Mount Moses, we use the ice surface elevation on the Larter Glacier side of the windscoop. At Wold Nunatak, we use the elevation of the ice surface near the base of the lowest exposed rock facing Larter Glacier. At Siren Rock, we use a break in the ice surface slope on the south side of the nunatak facing Larter Glacier. At Slusher Nunatak, we use the ice surface elevation at the base of a ridge extending towards the Lucchitta Glacier tributary. At Dean Nunataks Knoll, we use the ice surface elevation at the base of an outcrop on the side of the nunatak facing Lucchitta Glacier. Similarly, at Dean Nunataks Crater, we also use the elevation at the base of the nunatak facing Lucchitta Glacier. Local ice elevations and the locations used to define them are reported in Supplementary Data 6.

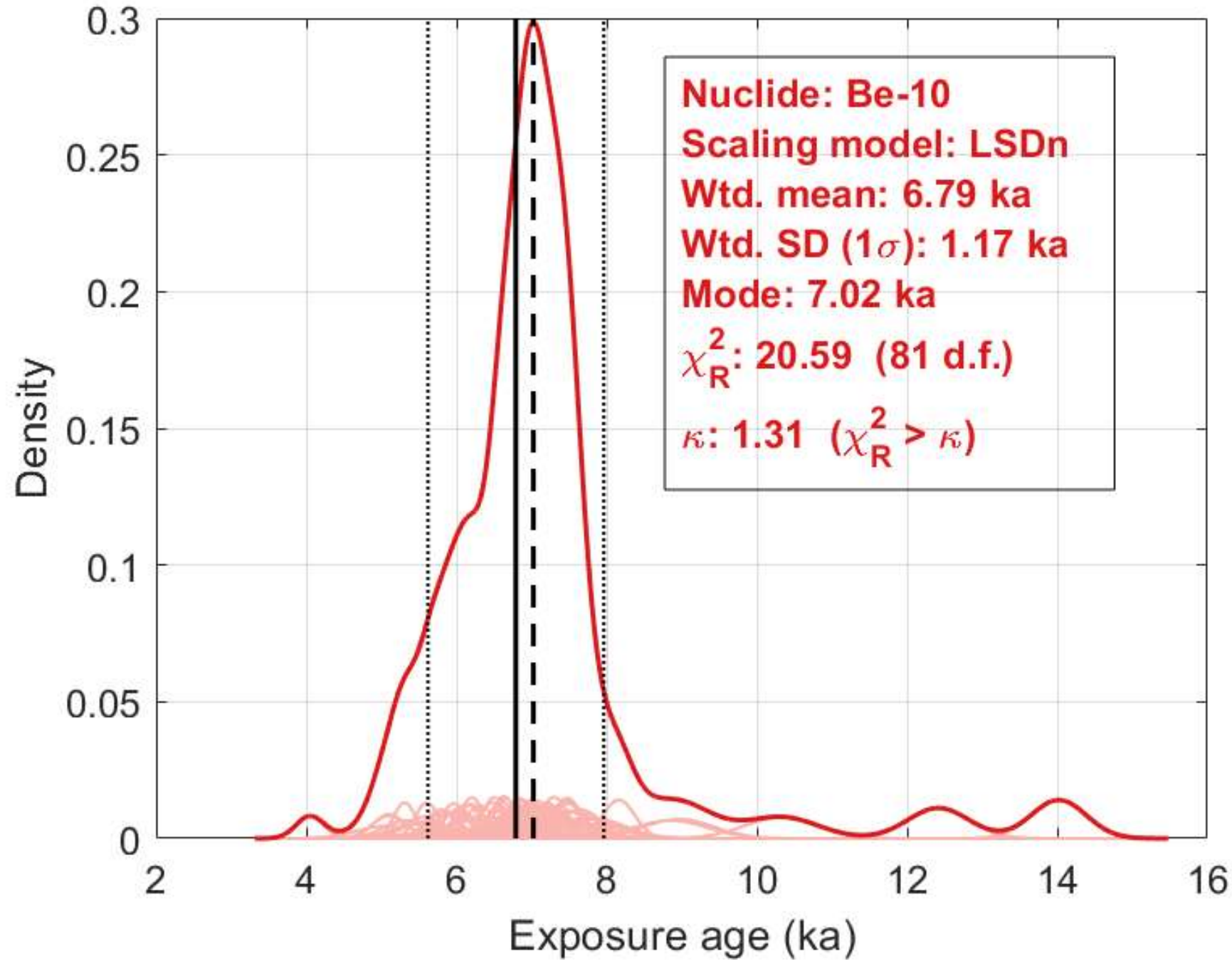

Supplementary Figure 1. Kernel density plot showing clustering of Hudson Mountains exposure ages. The plot was made using the MATLAB® model "iceTEA" (http://ice-tea.org)[9]. The dashed vertical black line shows the peak age density (mode; 7.02 ka) and the solid and dotted vertical black lines indicate the average exposure age with 1σ error (weighted mean; 6.79 ± 1.17 ka).

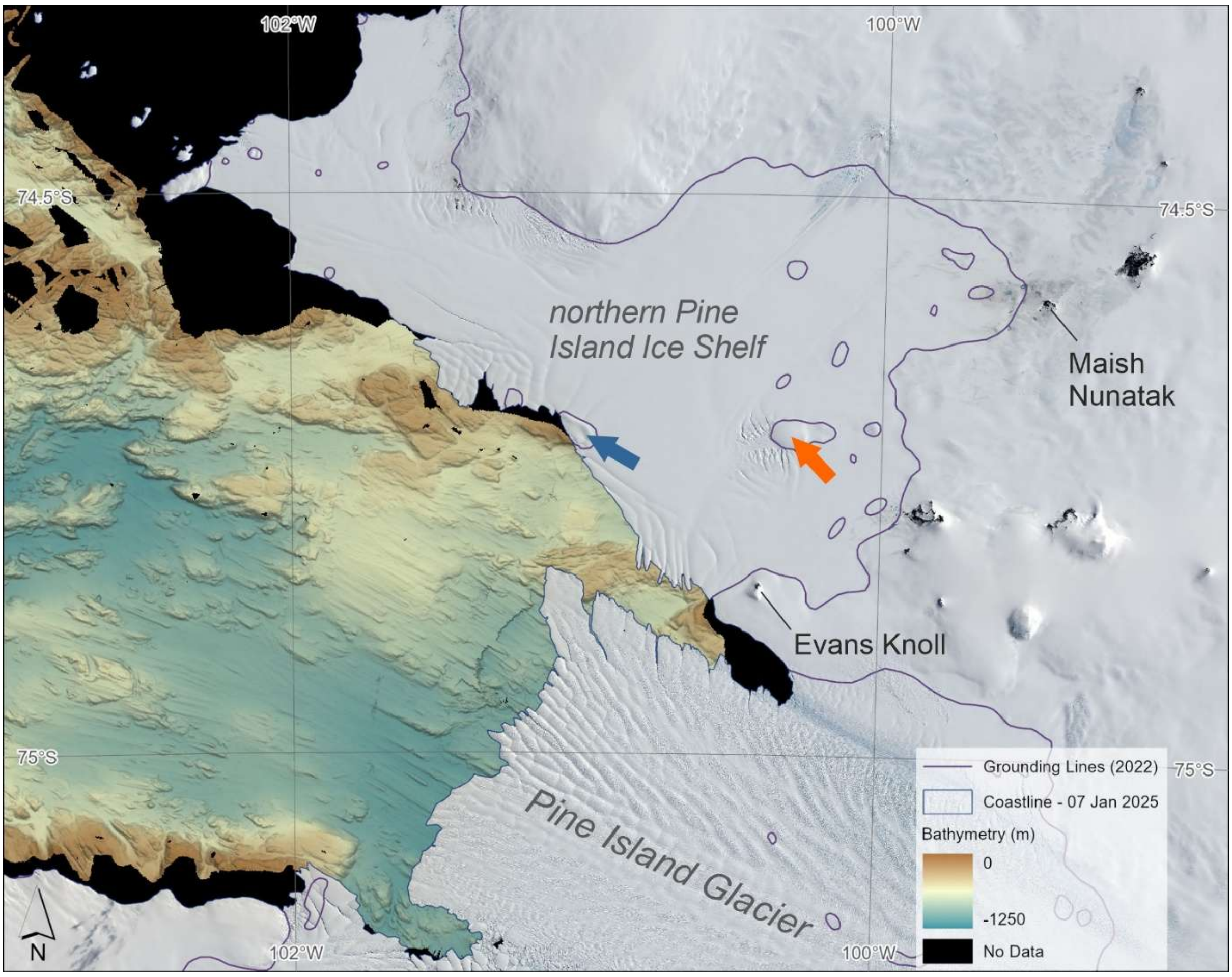

Supplementary Figure 2. Satellite imagery and bathymetry of inner Pine Island Bay. The satellite image (Landsat-8 image, acquired 7 January 2025; courtesy USGS) shows surface features in northern Pine Island Ice Shelf, including a large ice rumple (indicated by orange arrow) ~10 km seaward of the grounding line of Larter Glacier and a similar feature at the ice-shelf edge (indicated by blue arrow). The bathymetry data are from ref. 10 and unpublished data of Frank O. Nitsche (personal communication, 29 October 2025). The 2022 grounding line is from ref. 11.

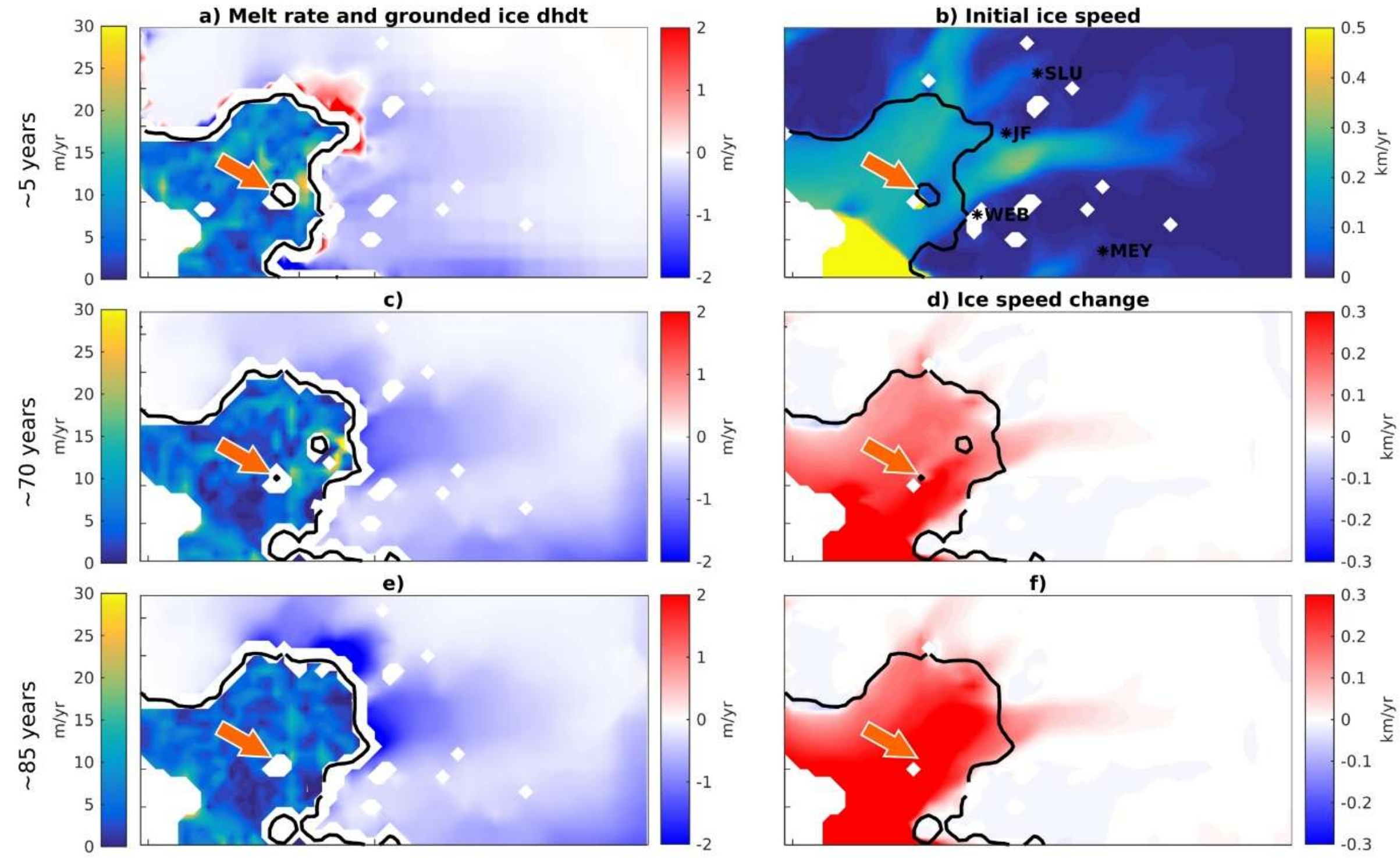

Supplementary Figure 3. Results of coupled ice-ocean model simulation of the Pine Island Glacier system[12]. Panels in the left-hand column show snapshots of ice-shelf melt rates and rates of grounded-ice thickness change (dhdt). Panels in the right-hand column show the initial ice speed (panel b) and snapshots of changes in ice speed (panels d and f). The coupled model setup and simulations are described in ref. 12 and the data are in ref. 13. Labels in panel (b) show selected nunatak locations as follows: SLU, Slusher Nunatak; JF, Maish Nunatak; WEB, Webber Nunatak; MEY, Meyers Nunatak. In the model simulation shown, initial ice-shelf melting induces unpinning of grounded ice from the large ice rumple currently located within the northern PIIS (location indicated with orange arrow) after ~70 years. This is followed by the creation and subsequent ungrounding of a new pinning point after ~85 years. The combined ungroundings are associated with grounding-line retreat, speed-up of Larter and Lucchitta glaciers and ice-sheet thinning upstream (for example in the Hudson Mountains, with an average rate of ~0.1 to ~0.4 m yr^{-1}). Unpinning occurs in both the “warm ocean forcing” scenario (shown here) and with “cold ocean forcing”; the warm and cold forcings bracket, and are idealised representations of, the warmest and coldest recorded ocean conditions in the Amundsen Sea embayment so far this century, corresponding to different thicknesses of the warm Circumpolar Deep Water layer reaching the base of PIG.

Site name	Number of data points (n)	Altitudinal range (m)	Best-fit average thinning rate (m yr^{-1})	Oldest exposure age (ka)	Youngest exposure age (ka)
**Observed rates:**					
Evans Knoll	4	79	0.13	8.2 ± 0.5	7.6 ± 0.5
Winkie Nunatak	6	55	0.07	7.7 ± 0.6	6.8 ± 0.5
Shepherd Dome	7	56	0.05	7.5 ± 0.5	6.0 ± 0.4
Inman Nunatak	5	10	0.02	7.3 ± 0.5	6.5 ± 0.4
Meyers Nunatak	4	28	0.01	10.3 ± 0.8	6.5 ± 0.4
Webber Nunatak	3	52	0.02	7.7 ± 0.7	6.2 ± 0.5
Mount Manthe	2	11	n.d.	8.9 ± 0.7	5.1 ± 0.4
Maish Nunatak	5	99	0.38	7.2 ± 0.5	6.8 ± 0.5
Mt Moses*	12	572	0.64	7.4 ± 0.6	5.3 ± 0.3
Mt Moses upper	*8*	*486*	*8.04*	*7.4 ± 0.6*	*6.6 ± 0.4*
Mt Moses lower	*4*	*86*	*0.05*	*7.0 ± 0.5*	*5.3 ± 0.3*
Wold Nunatak	8	115	0.09	7.0 ± 0.5	5.6 ± 0.5
Siren Rock	8	30	0.02	7.5 ± 0.6	5.6 ± 0.5
Slusher	11	79	0.05	9.0 ± 0.7	5.1 ± 0.5
Dean Nunataks	5	78	0.08	6.7 ± 0.5	5.1 ± 0.4
**Modelled rates:**					
Pulse 1	N/A	N/A	0.3252	N/A	N/A
Pulse 2	N/A	N/A	0.0344	N/A	N/A
Pulse 1	N/A	N/A	0.2374	N/A	N/A
Pulse 2	N/A	N/A	0.0655	N/A	N/A
Pulse 1	N/A	N/A	0.2489	N/A	N/A
Pulse 2	N/A	N/A	0.0702	N/A	N/A

Supplementary Table 1. Observed and modelled average ice-sheet thinning rates (m yr^{-1}) in the Hudson Mountains region. For sample sites, these are derived from Monte Carlo linear regression analysis of the exposure-age data presented in Supplementary Data 3, using the MATLAB® model "iceTEA" (http://ice-tea.org)[9]. Rates of ice-surface lowering were generated from 5000 iterations through randomly-sampled points using 2σ internal uncertainties. Data sites are listed in order of distance from the modern grounding line (closest first) for each glacier and coloured as follows: orange = PIG sites, purple = Larter Glacier sites, green = Lucchitta Glacier sites. The thinning rate for Mount Manthe was not determined ("n.d.") because there are insufficient data points (n=2). The modelled thinning

rates are reported here for each episode of thinning ('Pulse 1' referring to the earlier episode, 12.0-10.6 ka, and 'Pulse 2' referring to the later episode, 9.4-5.0 ka; Fig. 3), along each glacier coloured as for the data sites, and do not account for changes in the thinning rate through time. *The thinning rate at Mount Moses was previously reported using a two-segment age-elevation history[6]; for consistency with other sites, here we report the average *total* thinning rate but include rates for two segments for comparison.

Supplementary References

1. Nichols, K. A. et al. Offshore-onshore record of Last Glacial Maximum-to-present grounding line retreat at Pine Island Glacier. *Geology* **51** (11), 1033–1037 (2023). https://doi.org/10.1130/G51326.1
2. Howat, I. M., Porter, C., Smith, B. E., Noh, M. -J., Morin, P. The reference elevation model of Antarctica. *The Cryosphere* **13**, 665–674 (2019). https://doi.org/10.5194/tc-13-665-2019
3. Johnson, J. S. et al. Glacial geology of the Hudson Mountains, Amundsen Sea sector, West Antarctica. *Quat. Sci. Rev.* **350**, 109027 (2025). https://doi.org/10.1016/j.quascirev.2024.109027
4. Balco, G. et al. Reversible ice sheet thinning in the Amundsen Sea Embayment during the Late Holocene. *The Cryosphere* **17**, 1787–1801 (2023). https://doi.org/10.5194/tc-17-1787-2023.
5. Johnson, J. S., Bentley, M. J. & Gohl, K. First exposure ages from the Amundsen Sea Embayment, West Antarctica: the Late Quaternary context for recent thinning of Pine Island, Smith and Pope Glaciers. *Geology* **36 (3)**, 223-226 (2008). https://doi.org/10.1130/G24207A.1
6. Johnson, J. S. et al. Rapid thinning of Pine Island Glacier in the early Holocene. *Science* **343**, 999–1001 (2014). https://doi.org/10.1126/science.1247385
7. Nichols, K., Rood, D., Wilcken, K. & Johnson, J. Cosmogenic ^{10}Be data and calculated surface exposure ages from nunataks adjacent to Pine Island Glacier in the Hudson Mountains, West Antarctica (Version 1.0) [Data set]. NERC EDS UK Polar Data Centre (2023). https://doi.org/10.5285/9ddd50c8-cd08-4afe-b245-f6891f1d9a3f
8. Nichols, K., Rood, D., Johnson, J., Wilcken, K. & Brown, K. Cosmogenic ^{10}Be data and calculated surface exposure ages for 41 cobbles and boulders collected from nunataks adjacent to Larter and Lucchitta glaciers in the Hudson Mountains, West Antarctica (Version 1.0) [Data set]. NERC EDS UK Polar Data Centre (2025). https://doi.org/10.5285/de4a62bc-9c01-4aa3-9fd8-c64d706cfc13
9. Jones, R. S., Small, D., Cahill, N., Bentley, M. J. & Whitehouse, P. L. Tools for plotting and analysing cosmogenic-nuclide surface-exposure data from former ice margins. *Quat. Geochronol.* **51**, 72–86 (2019). https://doi.org/10.1016/j.quageo.2019.01.001
10. Hogan, K. A. et al. A multibeam-bathymetric compilation for the southern Amundsen Sea shelf, 1999-2019 (Version 1.0) [Data set]. UK Polar Data Centre, Natural Environment Research Council, UK Research & Innovation (2020). https://doi.org/10.5285/f2dfeda9-bf44-4ef5-89a3-ee5e434a385c

11. Moholdt, G., Pritchard, H., & Maton, J. RINGS/Bedmap3 grounding line of the Antarctic Ice Sheet [Dataset]. Norwegian Polar Institute (2025). https://doi.org/10.21334/NPOLAR.2025.D1062D2A.
12. Bett, D. T. et al. Coupled ice–ocean interactions during future retreat of West Antarctic ice streams in the Amundsen Sea sector. *The Cryosphere* **18**, 2653–2675 (2024). https://doi.org/10.5194/tc-18-2653-2024.
13. Bett, D. Amundsen Sea sector ice-ocean model output focused on pinning point ungrounding in the Pine Island Glacier system [Data set]. Zenodo. https://doi.org/10.5281/zenodo.21128994 (2026).
