# Supplementary material for: Synchronous Holocene thinning of Pine Island Glacier and its tributaries influenced by ice-shelf unpinning
Source: Nat Commun. 2026 Aug 11;17:9639. doi: 10.1038/s41467-026-76244-6 (PMC13554069; doi:10.1038/s41467-026-76244-6)
Supplement: Supplementary file 3 [file 41467_2026_76244_MOESM2_ESM.pdf]

Description of additional supplementary files

Supplementary Data 1:

All ^{10}Be exposure ages from Hudson Mountains (i.e. published ages, and new ages presented for the first time in this paper). [separate MS Excel spreadsheet]

Supplementary Data 2:

Sample information from new analyses required for exposure age calculation (latitude, longitude, elevation, topographic shielding correction factor, and sample thickness). [separate MS Excel spreadsheet]

Supplementary Data 3:

Analytical data and exposure ages (including $^{10}Be/^{9}Be$ ratios and ^{10}Be concentration) for the new analyses. [separate MS Excel spreadsheet]

Supplementary Data 4:

Procedural blanks for the new analyses. [separate MS Excel spreadsheet]

Supplementary Data 5:

Information formatted for online exposure age calculator input, for new analyses. [separate MS Excel spreadsheet]

Supplementary Data 6:

Relative ice-surface elevations and locations used to define them. [separate MS Excel spreadsheet]

Supplementary Movie 1:

Modelled thinning at nunataks in the Hudson Mountains through the last 15 kyrs. The left-hand panel shows rate of grounded-ice thickness change (dh/dt). Circles represent nunatak locations and are coloured according to the glacier whose thinning history they reflect (see text). Solid and dashed lines in the right-hand panel are the modelled ice-sheet thinning profiles at each nunatak, with the lines coloured according to the related glacier, as in the left-hand panel. [separate MP4 file]
